# BertSRC: transformer-based semantic relation classification

**DOI:** 10.1186/s12911-022-01977-5

**Published:** 2022-09-06

**Authors:** Yeawon Lee, Jinseok Son, Min Song

**Affiliations:** 1 Department of Library and Information Science, Yonsei University, Seoul, South Korea; 2 Department of Digital Analytics, Yonsei University, Seoul, South Korea

**Keywords:** Relation extraction, Semantic relation classification, Corpus construction, Annotation method, Deep learning, BERT, Fine-tuning

## Abstract

The relationship between biomedical entities is complex, and many of them have not yet been identified. For many biomedical research areas including drug discovery, it is of paramount importance to identify the relationships that have already been established through a comprehensive literature survey. However, manually searching through literature is difficult as the amount of biomedical publications continues to increase. Therefore, the relation classification task, which automatically mines meaningful relations from the literature, is spotlighted in the field of biomedical text mining. By applying relation classification techniques to the accumulated biomedical literature, existing semantic relations between biomedical entities that can help to infer previously unknown relationships are efficiently grasped. To develop semantic relation classification models, which is a type of supervised machine learning, it is essential to construct a training dataset that is manually annotated by biomedical experts with semantic relations among biomedical entities. Any advanced model must be trained on a dataset with reliable quality and meaningful scale to be deployed in the real world and can assist biologists in their research. In addition, as the number of such public datasets increases, the performance of machine learning algorithms can be accurately revealed and compared by using those datasets as a benchmark for model development and improvement. In this paper, we aim to build such a dataset. Along with that, to validate the usability of the dataset as training data for relation classification models and to improve the performance of the relation extraction task, we built a relation classification model based on Bidirectional Encoder Representations from Transformers (BERT) trained on our dataset, applying our newly proposed fine-tuning methodology. In experiments comparing performance among several models based on different deep learning algorithms, our model with the proposed fine-tuning methodology showed the best performance. The experimental results show that the constructed training dataset is an important information resource for the development and evaluation of semantic relation extraction models. Furthermore, relation extraction performance can be improved by integrating our proposed fine-tuning methodology. Therefore, this can lead to the promotion of future text mining research in the biomedical field.

**Supplementary Information:**

The online version contains supplementary material available at 10.1186/s12911-022-01977-5.

## Introduction

Biomedical literature is rapidly accumulating, and a large amount of this information is in the form of raw text, making it difficult to easily gather details on topics of interest. Instead of the researcher manually putting effort into collecting, reading, and understanding the literature, they can make use of text mining techniques, including text classification, clustering, topic modeling, and information extraction such as named entity recognition (NER) and relation extraction (RE). These can be effectively applied to the vast literature for automated information extraction, thus facilitating effective biomedical research processes [1].

Among the various biomedical text mining techniques evolving as a result of research achievements in the natural language processing (NLP) field, relation extraction is critical. Relation extraction is defined as extracting meaningful associations between entities in literature. There are several types of relation extraction, including semantic relations, grammatical relations, negations, and coreferences, depending on the focus and aim of the task [2].

Specifically, researchers in the biomedical field primarily focus on semantic relations to identify various relationships between bio-entities and to infer undiscovered knowledge. This perspective of information extraction motivates limiting our scope to semantic relation extraction. Semantic relation classification in the biomedical field enables the automatic extraction of relations between biomedical entities such as diseases, medications, chemicals, genes/proteins, or medical tests from a particular work. Therefore, new relationships can be inferred, allowing scientific hypotheses or new knowledge to be discovered or confirmed by identifying mechanisms of interaction between these entities or pathways to target materials; this in turn facilitates biological or new drug development research, biological database curation, drug repositioning, and clinical decision making [3, 4].

In the machine learning field, relation extraction is a classification task that predicts whether there is any semantic interaction between two entities (binary-class classification) or what type of relation the identified interaction belongs to among multiple predefined relation types (multi-class classification). Since classification is a type of supervised learning, in which a model is fitted on a labeled training dataset, relation extraction involves annotating unstructured natural language text with named entities and relations between them. However, manually constructing such a dataset takes a considerable amount of time and resources. Especially annotating a dataset with semantic relation information is more time-consuming and laborious than constructing a corpus annotated with named entities. Entity annotation is a task of simply recognizing bio-instances and categorizing them into their proper type, whereas relation annotation takes entity annotation as a prerequisite and determines the semantic interaction between two entities, which further relies on human judgment. Thus, building and sharing quality datasets annotated by experts is a significant contribution to the field of biomedical text mining.

Intending to promote relation classification research in the biomedical field, after reviewing the available benchmark relational corpora (usually for protein–protein interactions (PPIs)), this study presents our newly built corpus annotated with unique relation types on a meaningful scale. Then, to demonstrate the feasibility of our corpus, we built a Bidirectional Encoder Representations from Transformers (BERT)-based relation classification model, called BertSRC, trained on our dataset. Moreover, we proposed a new fine-tuning methodology regarding formatting input tokens for BERT, which is the second contribution of our study. The corpus for semantic relation classification and the BertSRC code are publicly available at https://github.com/tsmmbio/BertSRC.

### Construction of a training dataset annotated with semantic relations

Semantic relation classification in the biomedical field has been studied primarily as part of shared tasks aimed at evaluating and advancing NLP techniques. Currently, most prestigious datasets tagged with semantic relations are from these tasks, such as BioNLP shared tasks on the recognition of biological events, which introduced the BioNLP-09, 11, 13 event corpus, and BioCreative shared tasks on PPI extraction, which generated the BioCreative-II relation corpus [5]. In most of these corpora, the entities that mainly receive attention are genes/proteins, and much of the focus is centered around the relations between them [6, 7]. Examples of such PPI corpora include LLL, BioInfer, IEPA, and HPRD50 [1].

Although such benchmark corpora exist, it is not enough to objectively verify and compare many of the latest algorithms. Since it is not easy to build a new dataset manually from the scratch, several approaches to compensate for data shortages with relatively little effort have been suggested. Kanjirangat and Rinaldi [8] proposed the shortest dependency path (SDP) feature to effectively eliminate noise samples when augmenting data using distant supervision. Sentences were parsed into a tree structure and the dependency was calculated to obtain the SDP between the two entity mentions. Only features with SDP from entities were filtered and used as input to the model in the form of a triplet. This strategy effectively produced training data and the model performed well in biomedical relation extraction tasks with a precision of 0.65.

Li et al. [9] has constructed an event-centered PPI ontology (PPIO, PPI Ontology) that includes the temporal and spatial vocabulary to represent the biological context of PPI events. Six key pieces of information (interactor, biological process, subcellular location, etc.) were expressed by integrating other thesauruses or ontologies including Gene Ontology and Protein Ontology. Designing such ontologies not only helps interpret the context of biological PPIs in the literature but also facilitates subsequent data construction by being a useful tool for PPI annotation tasks.

These works show that securing a reliable dataset in sufficient quantities remains challenging in RE. Specifically, while algorithms are domain-independent, datasets are not, causing a chronic lack of data. In this regard, manually annotating a dataset for a particular domain is a high contribution to the study of text mining in that domain, which is biology in this paper.

Among various semantic relation corpora, only those annotated with binary relations are included as the main scope of our study. A binary relation is a type of relationship where a pair of two entities come as arguments. Since this type of relation is easy to understand and make use of, it is the corpus annotated with this type of relation that most current information extraction (IE) systems require. For this reason, we deliberately excluded complex relations or events where different levels of relations could be nested and become an argument for the other relation along with the named entities from the scope of our study. The table comparing several representative benchmark corpora annotated with binary relations is provided in Section A of Additional file 1. We gathered information about these corpora by reviewing each corpus or through the literature presenting or explaining the corpus [10].

According to Section A in Additional file 1, some corpora annotate only genes/proteins as entities, while others annotate other participating entity types exhaustively along with genes/proteins. With regard to relation type, there are some corpora that do not define separate relation types at all; in contrast, they only determine the presence or absence of a connection within the scope they have determined. Other corpora explicitly define relation types and express dynamic hierarchical relationships among them, forming complex ontologies. The former case has a limitation in that there is only limited utility for bio-researchers because various aspects of the relation between bio-entities and semantic relationships between those relations cannot be expressed. On the other hand, the latter has the advantage of being able to reflect detailed characteristics and meaning of relations. However, introducing many classes, such as in the case of BIOINFER, which has more than 60 relation types, requires a larger dataset, a more thoughtfully designed structure, and a complicated parameter tuning process in order to make a model generalize well. This could limit the applicability of the corpus compared with the dataset working with the model without much configuration.

Therefore, we present a new corpus annotated with semantic relation types differentiated from those of the existing corpora to provide researchers with useful and easily implemented resources for bio-text mining. Regarding entity, our corpus exhaustively covers many types including genes/proteins as long as they form meaningful relations. The relation is divided into two broader types according to the presence or absence of causality. If a causality doesn’t exist, it is considered an undirected link, which is a negative example, otherwise, a directed link. On the other hand, a directed link, which is judged to be causal, can be classified into a more subdivided type, such as a positive cause and negative cause, if the direction of causality is clearly revealed in the sentence. This hierarchical structure of relations has no biological meaning and is intended to introduce certainty about the interaction. It allows researchers to determine the reliability of the relation revealed in the text on their own because the relation type itself defines the level of its specificity and certainty.

### Deep learning-based semantic relation classification model

We evaluated the feasibility of the dataset that we built by constructing a semantic relation classification model and training it on the dataset. Furthermore, we sought to improve the performance of relation extraction by suggesting a new fine-tuning method. Since relation extraction has significant applications in various NLP tasks, improving the performance of relation extraction models can also improve the quality of various application tasks such as information extraction and knowledge graph construction.

Methods for implementing a relation classification task are largely divided into rule-based, statistical learning-based, and deep neural network-based, as in other information extraction studies such as NER and entity disambiguation [11]. Traditionally, rule-based and statistical-based machine learning has been widely used, but recently most NLP researches rely on a neural network model based on distributed features that do not require syntactic parsing or complicated feature engineering. Zeng [12] achieved state-of-the-art performance by applying CNN, a deep learning architecture mainly used for image and video processing, to a relation classification task, with only word and sentence level distribution vector as input, without discrete features, which were effective in the traditional machine learning methods. After this groundbreaking study, many studies have applied deep learning algorithms such as CNN, RNN, and LSTM for various NLP tasks[12–18]. Kim [18] constructed a plant-disease relations corpus and proposed a classification model trained on this corpus. They noted that Zeng’s model outperformed the SVM, which had shown the best performance in classification tasks so far by applying CNN, and then deployed CNN as the basic architecture of the model to verify the effectiveness of the constructed corpus, obtaining an f-score of 0.764.

Currently, one of the deep learning architectures, Google's Transformers [19], has replaced traditional rule-based, statistical-based NLP techniques as well as other deep learning architectures used in NLP's various tasks such as CNN, RNN, and LSTM. The current leading NLP models such as BERT[20], GPT[21], and T5[22] announced later are all based on this transformer block. In particular, BERT is commonly used in biomedical text mining research because it is built on multiple transformers encoder blocks, which has the advantage of compressing the sentence and mining semantic information from it [8, 23–25].

BERT's excellence in RE tasks stems from the fact that it is not only built on the transformer block which already has a proven track record, but also the context of the input sequence can be learned in both directions. In contrast to a language model for sequence generation such as GPT, a model that with a given sequence, predicts the next word, BERT is a masked language model that analyzes both directions of input in the pre-training stage to obtain embeddings for the language. By referring to the entire context of the sentence, it can produce vectors that can well reflect the semantic meaning of each token in the sentence. In other words, the quality of embeddings representing text is ahead of other models. Thus, implementing a model for NLP downstream tasks such as RE with these embeddings performs well [20]. Therefore, BERT is an ideal deep learning architecture for our study, which aims at predicting the semantic relations between bio-entities in biomedical literature.

Hong et al. [25] created a dataset labeled with predicate relations by performing automatic NER on SemMedDB data and then clustering on predicates that appear with the recognized entity pairs. Various deep learning models were trained with the dataset to verify the usability of their dataset and propose the final state-of-the-art performance model optimized with the dataset. Experiments demonstrated that the performance of BERT was better than that of CNN or LSTM, which had been widely used in the existing NLP research. Among BERT and its variant architectures, BioBERT and SciBERT which are specialized in biology and science literature showed excellent performance with f1-scores of 0.86 and 0.84, respectively. This is because BERT architectures, pre-trained on vast scientific literature, were able to learn sequential characteristics of text in the biomedical field.

Bioinformatics studies are also actively introducing BERT. Since the string sequence of the protein or gene has a structure similar to that of the natural language, NLP can be applied to analyze protein sequence. Using BERT, proper embeddings for the protein can be acquired which extract its sequence information well. To automatically identify sites of DNA 6 ma, Le et al. [26] generated embeddings of DNA sequences through BERT blocks and used the CNN structure in the classification layer that predicts whether it is 6 ma sites or not. The classification model performed better than other baseline models with an accuracy of 79.3% and MCC of 0.58. A similar study [27] also conducted a case study of identifying DNA enhancers from DNA sequence representations, using hybrid models of BERT and CNN. The identifying classifier used CNN, and the fixed vector for each nucleotide entering as input of this classifier was obtained through BERT. The BERT-based vectors have resulted in statistically significant improvements in sensitivity, acuity, and MCC than unidirectional word embedding features such as Word2Vec and fastText. These studies have shown that the combination of BERT and CNN has strength in modeling protein structures.

BERT is also being utilized in the medical and clinical fields to automatically analyze various medical data such as electronic health records [24, 28]. The need for a systematic review is emerging for evidence-based diagnosis and treatment in the medical field. However, it can be time-consuming for the medical staff to manually perform systematic reviews of numerous documents, resulting in outdated information. Therefore, automating SR with NLP technology is attempted [28]. BERT showed state-of-the-art performance in document classification and relation extraction tasks. In addition, experiments with different settings for BERT have been conducted to propose the best model with optimized performance. As a result, it was found that the size and class ratio of the training data play an important role in the model's performance.

As such, more recent studies applying NLP technology in a diverse domain, including the biofield, consistently demonstrate that the BERT algorithm based on the transformer structure performs very well, and in order to apply it to a specific domain, it is important to secure good quality data, which is the goal of our study.

In summary, we built a training dataset for semantic relation classification that annotates various bio-entity types and their semantic relational information. In addition, we proposed a new fine-tuning method for BERT to improve the performance of relation classification tasks. By comparing the relation extraction performance of models with various methodologies trained on the constructed dataset including the proposed methodology, we evaluated the usability of our constructed dataset and the performance of our proposed methodology. The creation of these novel datasets and fine-tuning methodology for classifying relations provides a meaningful contribution to this field and is expected to advance future semantic relation classification research.

The following parts of this paper are organized as is: Sect. 2. Material and Methods, sub-Sect. 2.1 reports on PubMed data collection and annotation procedures, including guidelines for building datasets. Section 2.2 presents setting details for constructing a classification model based on BERT architecture using the constructed dataset as training data. In addition, we present a fine-tuning methodology to enhance classification performance. In Sect. 3. Results, sub-Sect. 3.1 presents an overview of the constructed dataset, and 3.2 reports the results of the model comparison experiment and the structure of the best model. Finally, in Sect. 4, the conclusion and outlook of the entire paper are discussed.

## Material and methods

### Construction of semantic relation corpus

#### Data preparation

To begin with, PubMed data that were published between 2004 and 2019, a total of 15 years, were collected regardless of the topic within the boundaries of the biomedical field. A total of 1,500 candidate abstracts were obtained. After a brief inspection of the abstracts, we excluded documents that were too short or lacked sufficient entities for a relation to be assigned. The number of screened papers was 154, and a total of 1,346 filtered papers in our dataset were subject to annotations. The collected data were then separated into sentences, which became the unit of annotation in this work. Sentences were distinguished from one another based on the combination of the PMID that they belonged to and the sentence identity (ID) that represented the sequential order of the sentence in the text. The title of each work was treated as the sentence with the sentence ID of 0.

#### Annotation

We extracted different types of entities from the titles and abstracts of papers provided by PubMed and then built a semantic relation corpus that assigned semantic relation types to those entities based on the contextual information surrounding them and an expert’s judgment. Recognizing different types of entities outside the scope of proteins/genes and granting those entities semantic relations were highly dependent on manual expert curation. Such studies have not been extensively performed in the past.

Various bio-entities in each sentence were manually identified and annotated with the corresponding entity type. When annotating them or resolving any ambiguity after the annotation process, annotators referred to three databases, namely, Pubtator, Uniprot, and GeneCards, that contain information about bio-entities. Entity types that were annotated, with their definitions given later in the paper, included biological processes, cells, compounds, DNA, enzymes, genes, hormones, molecular functions, phenotypes, proteins, RNA, and viruses.

After the process of annotating entities was complete, the process of assigning the eight types of semantic relations between those annotated entities followed. The relation was decided based on the context of the sentence or the full abstract. The eight types of semantic relations included the following: undirected link, directed link, positive cause, positive increase, negative decrease, negative cause, positive decrease, and negative increase.

**Fig. 1:**
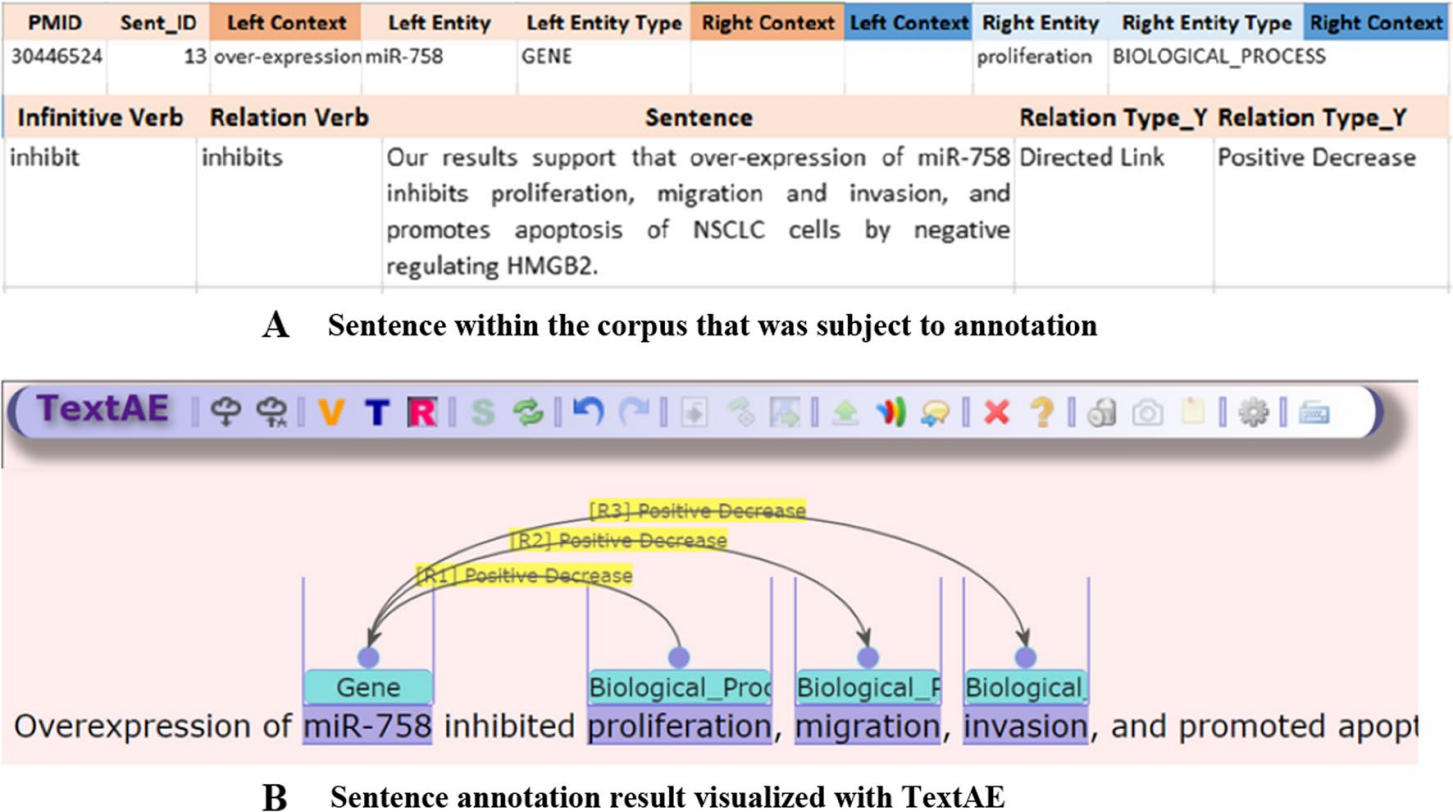
**A** Sentence within the corpus that was subject to annotation. **B** Sentence annotation result visualized with TextAE. Annotation tool based on TextAE: TextAE is a text annotation tool that can annotate named entity and relation information in the text. Each term (entity) can be dragged or double-clicked in *Term Edit Mode*, and the corresponding type can be selected to annotate them. If the corresponding entity type does not exist at the time, a new type can be defined. Likewise, in *Relation Edit Mode*, a type of relation can be selected or created and visualized. It is also possible to annotate multiple relations where one entity is associated with multiple other entities at once. The results of the annotation can be downloaded locally in the form of a json file

For the sake of convenience, the annotation work was assisted by an online annotation tool developed by our research team. The software tool is based on an open-source project TextAE (Text Annotation Editor),^1^ a visual annotation tool, that can support not only creating and saving annotation results but also conveniently retrieving and editing existing ones. This tool saved us significant time whenever we needed to revise the annotation guideline or amend previously accumulated annotations. The tool can be accessed at http://165.132.151.153/annotator/. Screenshots of the tool are given in the figure below (Fig. 1).

#### Annotation guidelines

##### Entity annotations

Recognition of named entities is a prerequisite in the relation extraction process. Discovering the relationship between two entities is possible after they are accurately recognized. To ensure accurate and consistent annotation, multiple annotators worked independently at first, and in the event of a discrepancy, a consensus was reached after multiple agreements to resolve it. Brief explanations of each entity type are shown below (Table 1). Please see Section B in Additional file 1 for details.

**Table 1:** Twelve entity types

	Explanation
Genes, DNA, RNA, and Proteins	A gene is the functional unit of heredity and the nucleotide sequence of DNA or RNA that holds instructions for synthesizing either RNA or protein
Enzymes	Proteins that act as biological catalysts
Hormones	Signaling molecules that act distant from their site of production
Compounds	Additives such as drugs or chemicals
Molecular functions	Proteins with the role of binding such as hormones and antigen-antibodies
Phenotypes	Limited to human diseases
Biological processes	Processes/activities that occur within cells
Cells	Smallest functional units of an organism with which biological experiments are conducted to observe their mechanisms or to grow targeted compounds
Viruses	Used to indicate when experiments are conducted on a virus

If more than one entity was recognized in a sentence, the sentence moved to the step of assigning the type of semantic relation between the entities.

##### Semantic relation annotations

Relation annotation is performed on two entities that appear within a sentence. In principle, the assignment of relations is based on the sentence in which the entities appear, but when ambiguous, the entire abstract of a piece can be read, and the annotator can decide based on the full context in the final stage. To define semantic relations between entities, we extracted relational verbs and contextual information between two entities. When determining semantic relations between entities, contextual information must be considered along with the meaning of the verb. For example, if “miR-194,” “basal and insulin-stimulated glucose uptake,” and “glycogen synthesis” were recognized as named entities in the sentence “Knockdown of miR-194 in L6 skeletal muscle cells induced an increase in basal and insulin-stimulated glucose uptake and glycogen synthesis” [29], the verb associated with “miR-194,” “basal and insulin-stimulated glucose uptake,” and “glycogen synthesis” would be “induce.” Although the verb “induce” is usually classified as positive, the word “knockdown of” followed by “miR-194” means inhibition; therefore, it should be classified as negative rather than positive. Thus, contextual information, which plays an important role in correctly classifying relations, should be annotated together.

The semantic relations defined in this study were classified into a total of eight types: undirected link, directed link, positive cause, positive increase, negative decrease, negative cause, positive decrease, and negative increase (Table 2). They were structured into three layers, and each level represents the extent of granularity. Initially, a relation between two entities in a sentence was classified into an undirected link and a directed link at the top level based on whether the causal relationship between the two entities was clearly revealed. When a relation was identified as a directed link where there was a causal relationship based on the sentence, if it could be decided whether the two entities were positively or negatively correlated, the relation proceeded down one level down and was matched with finer types (positive cause/negative cause) or stopped at the first level (directed link). Similarly, at the second level (positive cause/negative cause), if the sentence captured causality according to an increase or decrease in the amount of each entity or its strength with explicit expressions of quantity, the relation proceeded down to the lowest level (positive increase/negative decrease, positive decrease/negative increase) or stopped at the second level (positive cause/negative cause). With regard to the relation of an undirected link, which is a correlation without causation revealed, since this type of relation is rarely a subject of attention for researchers, it does not need to be broken down to a more granular level, and this type of relation was treated as a negative example. This stratified structure between the types allows researchers to determine the reliability of the relation revealed. In addition, depending on the purpose and usefulness of the classification, it can be easily converted to binary class and integrated with other benchmark datasets, which is commonly binary. In summary, with respect to the relationships that researchers might be interested in, each relation was classified into the most detailed and specific type possible. Examples of sentences corresponding to each relation type and detailed descriptions are provided in Section C of Additional file 1.

**Table 2:** Eight types of relations

Causality	Direction of causality	Expressions of quantity
Directed Link	Positive Cause	Positive Increase
		Negative Decrease
	Negative Cause	Positive Decrease
		Negative Increase
Undirected Link	–	

#### Annotation procedure

We finished collecting data in February 2019 and performed trial annotations until April 2019. During the trial period, entities and relations were annotated for a small amount of collected data referring to previous related works for exploratory purposes. At this time, entities were pre-annotated through an automated biomedical NER/RE system called PKDE4J [30], and annotators had to modify incorrect entity annotations or add missed annotations. Relations between these readily annotated entities were classified manually by four to six annotators. However, as the decision to include gene-related entities in our annotation scope in a more exhaustive way was made, the method of entity annotation also shifted from utilizing PKDE4J to manual annotation to cover entity types that PKDE4J is not aimed at extracting, such as enzymes and viruses. Throughout the manual annotation process, the lead annotator, a biology expert with rich hands-on experience in bio-corpus construction projects, refined the guidelines and detailed the workflows.

The full-scale annotation process based on the final guidelines and workflows began in May 2019. The annotators included the lead annotator and eight researchers working on text mining in the biomedical field. More specifically, two of the eight researchers were selected as senior annotators to coordinate the entire annotation process among the multiple annotators and played important roles in settling any ambiguities, such as mediating disagreements that failed to be resolved in the previous stage. The lead annotator controlled the final verification and resolved any remaining ambiguous cases.

As we decided to annotate both entities and relations manually from the beginning, we developed a web-based annotation tool to streamline the annotation process and introduced this tool to our task in earnest starting in June. The annotation process was completed at the end of June 2020. The entire process, including data preparation, took approximately one and a half years to complete.

**Fig. 2:**
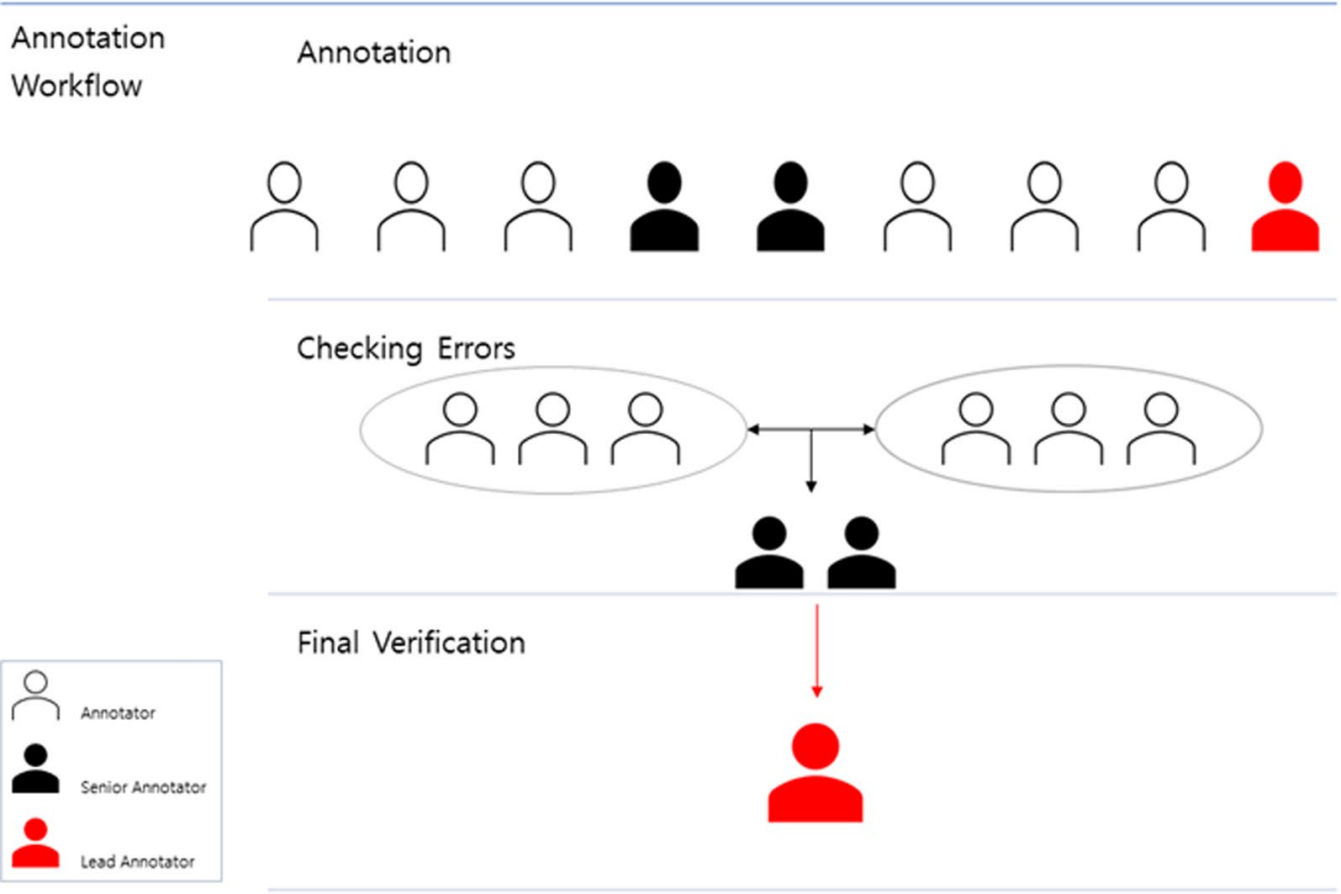
Annotation workflow

The final annotation workflow consisted of three stages: *annotation*, *error review*, and *final verification* (Fig. 2).

i. Annotation
  Annotators who had conducted research in the field of text mining and had experience building corpora in the biomedical field read the abstracts and manually annotated bio-entities by referring to the Uniprot, GeneCards, and Pubtator databases. They selected sentences in which two or more entities appeared and annotated the verb between the two entities and other contextual information that could help resolve any ambiguities. Based on this information and the annotator’s judgment after reading the sentence, the relationship between two entities was mapped into one of the eight semantic relation types we defined in this work.
ii. Error review
  Except for the lead and senior annotators, six annotators formed two teams of three to assess for simple errors within their own teams. If there was a disagreement within the teams, they attempted to reach an agreement in a reasonable direction through discussion and provision of evidence. The discussion usually resolved the disagreement. If the issue persisted, however, the teams convened to discuss the issue and moved forward with an agreement or passed the disagreement on to the senior annotators.
  The annotation result, which had undergone the first error review process, was delivered to the remaining two senior annotators who did not belong to either of the teams, and they conducted a second review process. The delivered annotation modifications and unresolved discrepancies were reviewed once more by these senior annotators, and agreement was attempted. Any inconsistencies that were not settled were finally resolved in the next verification step.
iii. Final verification
  To increase the quality of the dataset, the lead annotator verified the annotation result one last time in consideration of the context of the original text based on his or her biological knowledge. The lead annotator then corrected the result if necessary and decided whether to include it in the final dataset. The lead annotator not only adjudicated disagreements that could not be settled in the previous steps but also reviewed the entire dataset, including data that were already agreed upon by the teams for final verification. In practice, only a few simple errors were found after the multi-step review process, and these were quickly corrected.

Annotations that remained ambiguous even in the final verification stage were excluded from the final corpus.

### Semantic relation classification model training

To verify the credibility and usefulness of the semantic relation corpus we established, we attempted to build a semantic relation classification model that utilized the corpus. In order to fully optimize performance for our model, we compared the performances of several deep learning-based pre-training models and suggested new fine-tuning techniques for the model that produced optimal results. While performing the classification task, precision, recall, and f1 score are commonly used as performance metrics, so we adopt them to evaluate our model. The explanation and calculation formula for each metric are as follows (Table 3).

**Table 3:** Evaluation metrics

$Accuracy = \frac{{{\text{correct}}\;{\text{predictions}}}}{{{\text{total}}\;{\text{predictions}}}}$	Useful when target classes are well balanced
$Recall = \frac{True\;positives}{{{\text{True}}\;{\text{positives}} + {\text{False}}\;{\text{negatives}}}}$	The ability of a model to find all relevant cases within a dataset
$Precision = \frac{True\;positives}{{{\text{True}}\;{\text{positives}} + {\text{False}}\;{\text{positives}}}}$	The ability of a model to identify only the relevant data points
$F1 - score = \frac{{2\left( {precision \times recall} \right)}}{precision + recall}$	Combination between Precision and Recall Used to punish extreme values

In particular, all of the deep learning models used in our study are BERT-based architectures that are available through the well-known library Hugging face or are presented in precedent studies. As mentioned earlier, BERT is good at compressing and understanding the meaning of the text, making it suitable for our purpose, which is to extract semantic relations. Along with that, it is easy to implement and capable of making predictions immediately without much configuration, which is an important consideration to our work since the primary focus of the study was to demonstrate the feasibility of the corpus that we had constructed. For these reasons, we choose BERT as the model architecture to apply our data.

#### Pre-training and Fine-tuning stage of BERT

BERT is a pre-trained model that leverages the structure of the transformer encoder [19, 20]. Pre-trained models learn and utilize universal text embeddings rich in grammatical and semantic features from pre-training on a vast amount of textual data, and only a simple additional layer is needed for the aiming task. The basic BERT model was trained on the Book corpus (800 M words) and Wikipedia (2.5B), achieving SOTA in most common NLP tasks [20]. Since then, its variant models, which were pre-trained on domain-specific data, such as BioBERT [31], SciBERT [32], and PubMedBERT [33] as well as advanced versions of BERT with tweaked pre-training methods or a structure of layers, such as ALBERT [34] and RoBERTa [35], have been announced.

In the pre-training stage of BERT, a masked language model (MLM) and next sentence prediction (NSP) were utilized to learn various characteristics of natural languages [20]. The MLM method is a method of randomly replacing 15% of tokens with [MASK] tokens in the input sentence, expecting the BERT model to predict the original word of the [MASK] token. The pre-training of BERT involves two different sentences divided by the [SEP] token as input. At this time, 50% of the sentence pairs are in order, with the next sentence being the actual sentence that follows the prior in the original text, and the rest are not, with the first sentence being followed by a random sentence. NSP involves models learning to determine whether these two statements are in order. To develop a BERT model trained with these methods in the pre-training stage to perform downstream NLP tasks, such as relation extraction, an additional layer for detailed tasks is appended after the transformer encoder layer which has learned weights, and further fine-tuning is performed using relevant data for the desired tasks. This is how fine-tuning can provide a model for handling detailed downstream tasks.

During the fine-tuning stage, the simplest approach to processing input text for the relation classification task is using a single sentence containing the relationship between entities without any pre-processing treatment. However, to achieve optimal performance, introducing a slightly more complex input data processing method is necessary. Soares et al. [36] compared several fine-tuning methodologies for relation extraction, such as input structure, architecture of the downstream layer, a training setup, and explored effective ways to produce good-quality output vectors that represent relation for a given sentence.

#### Masked input

Yang et al. [37] demonstrated that there is a mismatch in the original BERT model. The [MASK] tokens are used in the pre-training process but not in the fine-tuning stage. To compensate for this limitation, in this work, we use a methodology that utilizes [MASK] tokens as the input sequence in both the pre-training and fine-tuning stages for relation classification.

In the pre-training of BERT, input sentences containing [MASK] tokens are received as input data, and the original tokens for the [MASK] tokens within each sentence are predicted using the output of the final layer corresponding to the location of [MASK] tokens. Namely, the output of the final layer at [MASK] token contains contextual information needed to predict the original token replaced with [MASK]. Likewise, during the fine-tuning process, if the token corresponding to the entities of interest within the input sentence is replaced with a [MASK] token, the final output layer at the location of the replaced tokens can be considered to output a semantic and contextual vector for the token.

**Fig. 3:**
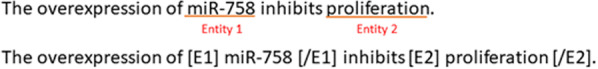
Entity marker–entity start: Input sentence with additional marker tokens

**Fig. 4:**
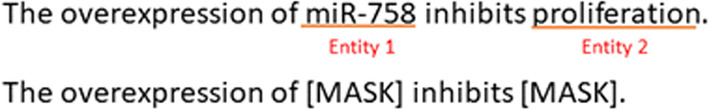
Masked input: Input sentence masked as [MASK] for entities

There are several objectives to using this approach. The first is to maintain consistency between the pre-training and fine-tuning training of the model. As mentioned above, the pre-training process for BERT employs [MASK] tokens that are not introduced in the fine-tuning stage, resulting in the disadvantage of inconsistent models. In this work, the [MASK] tokens are also utilized as input data in the fine-tuning process to increase the consistency of the model. The second objective is to effectively convey to the model the information of entities that span over multiple tokens. In relation classification, each entity often consists of multiple words. To deal with this problem, the aforementioned study by [36] introduced the method *entity marker–entity start*, which employs additional marker tokens, such as [E1], [/E1] and [E2], [/E2], before and after the entity to convey information about where the entity is located in the sentence. However, it still has the disadvantage of inconsistency because the tokens are not used for pre-training and the entity itself is not replaced (Fig. 3). Although this method enables the model to learn the span of entities in a given sentence, the model is limited in accurately recognizing additional marker tokens that were unseen in pre-training. Furthermore, it fails to directly convey information about the entities as a whole to the model. On the other hand, when replacing an entity itself with a [MASK] token, as suggested in this study, the entity exists as a token, and the output vector corresponding to the token contains contextual information that helps to effectively predict the meaning of the original word for the token (i.e., an entity) (Fig. 4).

Finally, it can alleviate the out-of-vocabulary (OOV) problem that occurs when a token that the model did not learn during training is introduced as input data. This problem can be improved using word piece tokenizing with BERT [20] but is not fully resolved. Tasks including named entity recognition and relation classification are likely to cause OOV problems because entities often contain proper nouns that have many variations of case or abbreviation (e.g., BERT, Bert, bert, bert algorithm). If an OOV problem occurs and the model cannot recognize entity tokens in a sentence correctly, it may struggle to predict the relation type between the entities. By replacing the entity with the [MASK] token, the OOV problems can be more effectively prevented.

However, the masked input method has a fatal drawback in that it loses the original token information of the entity. Therefore, we propose the *two masked sentence input* method, the masked input method coupled with the two-sentence input method, to overcome this weakness.

#### Two-sentence input

**Fig. 5:**
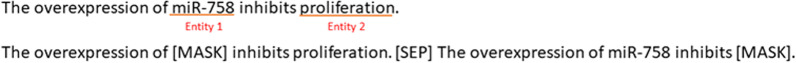
Two masked sentence input

Two-sentence input is a method of utilizing two identical sentences that are linked with [SEP] tokens as input data in fine-tuning. In this paper, specifically, we propose the *two masked sentence input* method, which masks one of the two entities in each of the sentences (Fig. 5). The masked entities are different from each other to keep one of the original entities unmasked, which is more beneficial than simply linking the duplicate of the sentence.

The first advantage of this method is that it maintains consistency in the pre-training and fine-tuning stages. In the original BERT paper, the pre-training stage exploited two sentences linked with the [SEP] token. During fine-tuning for the classification task, however, only one sentence was used, which can be disadvantageous to the performance due to the fact that the model’s learning process is inconsistent. The second advantage is that this method prevents information loss associated with the masked input methodology. Within sentences that have multiple entities, we can preserve token information by replacing only one entity at a time with the [MASK] token. This method completely prevents the loss of token information that can be caused by using masked input. Finally, this method conveys sequential information about the entity to the model. In relation extraction tasks, the relationship might be decided differently if the order of the first entity and the second entity are reversed. Therefore, it is critical that the model accurately recognizes the order of entities. Soares [36] used additional numbered marker tokens to carry sequence information to the model, but there is a limit to the model’s recognition of additional tokens that have been unseen during pre-training. In our two masked sentence input method, the first entity is replaced with the [MASK] token in the first sentence, and the second entity is replaced with the [MASK] token in the second sentence, effectively passing the semantic and contextual information corresponding to the first and second entities to the model in order.

**Fig. 6:**
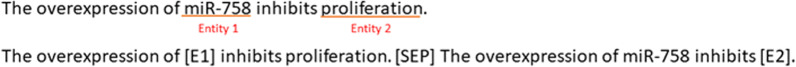
Two-sentence entity token input

Additionally, for multilateral comparison, we included another variant method—named the *two-sentence entity token input* method— in the comparison experiment (Fig. 6). This is a combination of the *entity marker–entity start* and the *two masked sentence input*, which is replacing two entities in a sentence differently with additional entity tokens, [E1] and [E2]. Since this is also a two-sentence input strategy, the two replaced tokens for each entity are differentiated from each other.

#### Downstream layer structure

We also make a slight modification to the standard BERT downstream architecture for classification from the original BERT paper [20] which uses a special token [CLS]. Instead of mapping the entire input sequence to only a [CLS] special token, we produce two vectors corresponding to the location of the entities, to acquire semantic representations for each entity. With these vectors as input, the added classification layer optimizes the weights for determining relations between entities. This method is suggested in [36] and turned out to have the advantage of more effective learning for relation prediction by directly utilizing vectors corresponding to information of entities.

For comparison, we experimented with the conventional [CLS] token vector method, the aforementioned method that utilizes both vectors corresponding to entities, and the method that uses those two vectors along with the [CLS] token vector altogether. We call these three methods respectively CLS token layer, two-token layer, and three-token layer.

In the CLS token layer, the input size of the classification output layer is set to 512, which is the output size of each layer of the pre-trained BERT model. In the two token layer, the input size is set to 2 × 512 (1024), which is the size of two outputs combined. In the three-token layer, which receives three BERT layer outputs as input, the input size is set to 3 × 512 (1536), which is the size of three outputs combined. The output size of the classification layer for all the methods is set to 8, which is the number of relation types or the number of classes to be predicted. The loss function of the model is cross-entropy loss.

Our three main experiments–comparison of pre-trained BERT models, comparison of masking input methods, comparison of classification–use each model’s PyTorch implementation by HuggingFace. AdamW was used as an optimizer in all models, and the performance was reported by applying the best-performing model to the test data after training up to 10 epochs. The commonly applied hyperparameters are shown in Table 4.

**Table 4:** Hyper parameters

Hyper Parameter	
num_train_epochs	10
learning_rate	5e-5
per_device_train_batch_size	16
per_device_eval_batch_size	64
warmup_ratio	0.1
weight_decay	0.01
adam_beta1	0.9
adam_beta2	0.999
adam_epsilon	1e-8
max_grad_norm	1

**Fig. 7:**
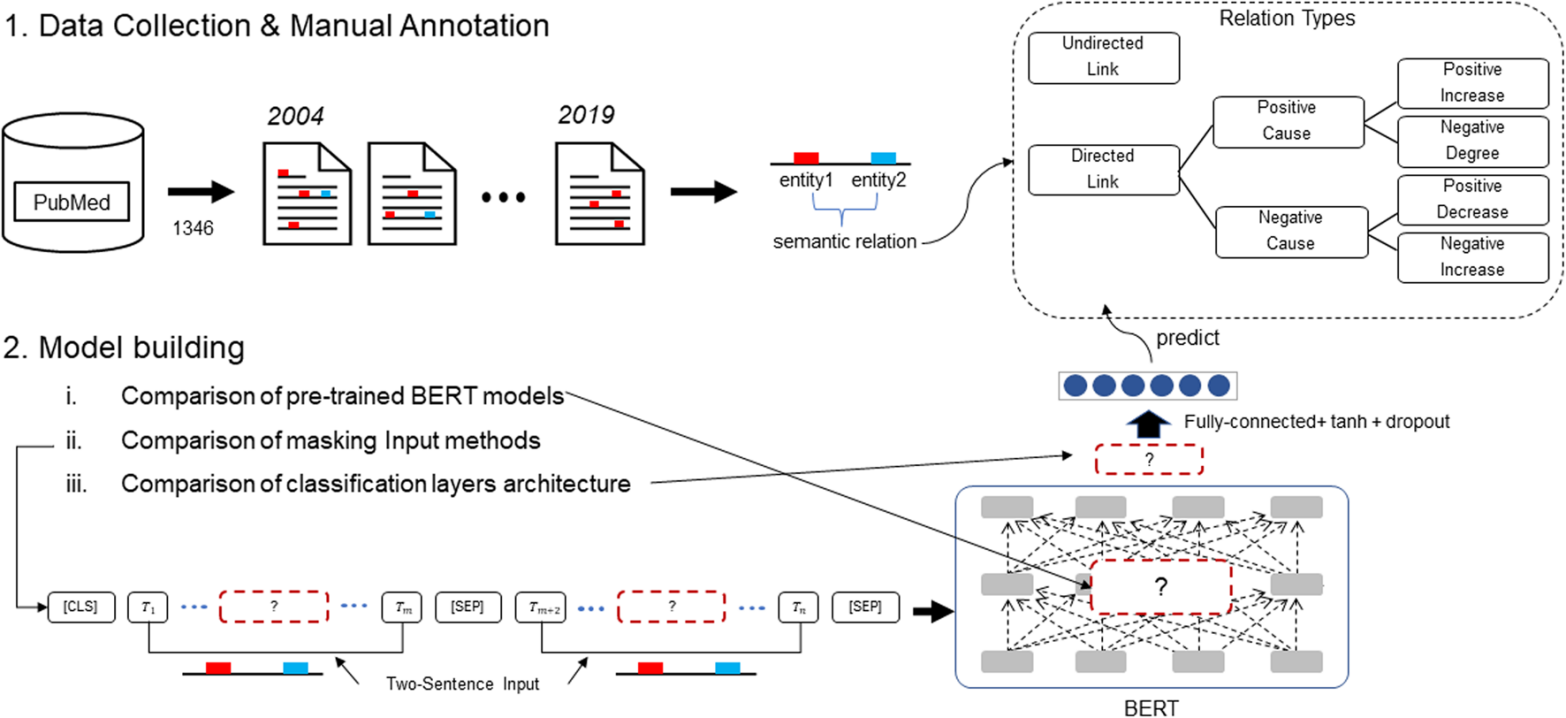
The overview of the study

The overview of the study is illustrated in Fig. 7.

## Results

### Dataset overview

We constructed a semantic relation corpus consisting of 1,346 abstracts annotated with 5,031 relations classified into eight types. A total of 10,062 distinct bio-entities of 12 types were annotated with half of then in the left portion of the sentence and the rest in the right portion of the sentence. The general statistics for the corpus are shown in the table below (Table 5).

**Table 5:** **A** Entity types. **B** Relation types. Dataset overview

	Train		Validate		Test		
	Left	Right	Left	Right	Left	Right	
A							
BIOLOGICAL PROCESS	181	825	61	301	70	270	1,708
CELL	60	25	15	12	16	12	140
COMPOUND	769	225	232	75	266	72	1,639
DNA	4	1	4				9
ENZYME	67	34	28	8	20	17	174
GENE	1,017	598	347	180	328	185	2,655
HORMONE	25	10	6	3	7	4	55
MOLECULAR FUNCTION	59	71	24	26	21	20	221
PHENOTYPE	494	1053	168	355	160	368	2,598
PROTEIN	241	142	84	33	84	46	630
RNA	91	33	34	13	33	13	217
VIRUS	10	1	3		2		16
	3,018	3,018	1,006	1,006	1,007	1,007	10,062

	Train	Validate	Test	
B				
Directed Link	484	174	175	833
Negative Cause	403	141	156	700
Negative Decrease	208	82	74	364
Negative Increase	145	52	48	245
Positive Cause	662	219	206	1087
Positive Decrease	89	18	26	133
Positive Increase	172	54	47	273
Undirected Link	855	266	275	1396
	3018	1006	1007	5031

The verb in the sentence is one point of reference when classifying the relation type; however, it does not exclusively determine the relation type. A relation type is determined by comprehensively considering the verb and verb-related information in the sentence, contextual information around entities, and human interpretation. For example, common context words such as “inhibition” and “decreased” can reverse the meaning of a verb. Thus, the semantic relation type assigned to the sentence might be the opposite of the original meaning of the verb.

### Semantic relation classification model

#### Performance comparison between pre-trained models

While applying an effective masking methodology for input sentences and downstream layers to the model is crucial, it is also important to select a pre-trained language model that best fits our tasks and data as the base model. Therefore, we compared the performance of various existing pre-trained BERT models for our dataset with the same hyperparameters specified in Table 4, setting two masked sentence input and two-token layer for all models. The models were evaluated using fivefold cross-validation with all of the train, validation, and test sets combined (Table 6).

**Table 6:** Performance comparison of pre-trained language models

Model	Accuracy	Precision	Recall	F1-score
BERT [20]^a^	*0.849 **(0.003)	0.817 (0.010)	0.822 (0.019)	0.818 (0.011)
BioBERT [31]^b^	0.861 (0.008)	0.835 (0.017)	0.846 (0.015)	**0.839 (0.011)**
**PubMedBERT[34]**^**c**^	**0.865 (0.014)**	0.833 (0.020)	**0.849 (0.009)**	**0.839 (0.015)**
RoBERTa [35]^d^	0.862 (0.009)	0.835 (0.018)	0.837 (0.009)	0.835 (0.010)
SciBERT [32]^e^	0.862 (0.010)	**0.836 (0.017)**	0.843 (0.013)	0.838 (0.013)

The best scores are in bold

^*^Mean

^**^Standard deviation

^a^Bert-base-uncased, Accessed July 20, 2022, Available from: https://huggingface.co/bert-base-uncased

^b^Biobert-base-cased-v1.2, Accessed July 20, 2022, Available from: https://huggingface.co/dmis-lab/biobert-base-cased-v1.2

^c^BiomedNLP-PubMedBERT-base-uncased-abstract-fulltext, Accessed July 20, 2022, Available from: https://huggingface.co/microsoft/BiomedNLP-PubMedBERT-base-uncased-abstract-fulltext

^d^Roberta-base, Accessed July 20, 2022, Available from: https://huggingface.co/roberta-base

^e^Scibert_scivocab_uncased, Accessed July 20, 2022, Available from: https://huggingface.co/allenai/scibert_scivocab_uncased

Comparisons have shown that PubMedBERT models pre-trained on abstracts from PubMed and full-text articles from PubMedCentral performed better than others. This confirms that when building a downstream model using a pre-trained language model, the data used for pre-training should be homogeneous to those used for fine-tuning. Therefore, PubMedBERT, which performed the best on our PubMed datasets, will be used as the base model in later experiments.

#### Performance comparison between methods of masking input

For this experiment, we used PubMedBERT, the language model with the best performance in the abovementioned comparative experiment, as the base model and the CLS token layer as the downstream output layer. The methods were evaluated using fivefold cross-validation with all of the train, validation, and test sets combined (Table 7).

**Table 7:** Performance comparison of masking input methods

Method	Accuracy	Precision	Recall	F1-score
Default	*0.700 **(0.018)	0.646 (0.019)	0.647 (0.016)	0.642 (0.013)
Entity Marker–Entity Start	0.857 (0.012)	0.825 (0.029)	0.836 (0.007)	0.828 (0.015)
Masked Input	0.844 (0.013)	0.811 (0.023)	0.826 (0.011)	0.817 (0.012)
**Two Masked Sentence Input**	**0.866 (0.014)**	0.837 (0.018)	**0.847 (0.013)**	**0.840 (0.014)**
Two Sentence Entity Token Input	0.865 (0.012)	**0.839 (0.019)**	0.845 (0.014)	**0.840 (0.015)**

The best scores are in bold

^*^Mean

^**^Standard deviation

We specifically compared the performance of the following methods: default method, which only adds [CLS] token in front of a sentence, entity marker–entity start, which marks a span of entities, masked input, two masked sentence input, and two sentence entity token input, which masks entities with additional tokens other than [MASK].

The comparison of input methods showed that the two sentence entity token input and the two masked sentence input methods, which used two combined sentences as input data, performed better than default method, the entity marker–entity start method or the masked input method. Two masked sentence input, which replaces entities with [MASK] tokens, was superior to using additional tokens, [E1] and [E2] tokens, as a way to replace entities.

These findings show that leveraging [MASK] tokens is better than introducing additional tokens such as [E1] and [E2] to replace entities. This confirms that maintaining consistency between the pre-training and fine-tuning stages can lead to improved performance. Furthermore, [MASK] tokens, which have only been used in pre-training phases, can be appropriately utilized in downstream tasks. In addition, the two sentence input methodology performed better than the methodologies where only one sentence is entered as input; this finding suggests that in such a relation classification task, using two identical sentences, each of which contains a masked entity, can lead to improved performance.

#### Performance comparison of downstream layers

Additional downstream layer construction is essential to fine-tuning a model for specific NLP tasks, using pre-trained models. Relation extraction models based on BERT require the addition of the classification output layer for relation prediction after the output of the transformer encoder. For performance comparisons between different layer structures, we equally apply the two masked sentence input methodology to the same PubMedBERT model with the same hyperparameters as the previous experiments. In this experiment, we compare the performance of the CLS token layer, using the output of the [CLS] token location, two-token layer, using the output values of the two [MASK] token locations, and three-token layer, using the output values of the [CLS] and two [MASK] token locations. The layer architectures were evaluated using fivefold cross-validation with all of the train, validation, and test sets combined (Table 8).

**Table 8:** Performance comparison of downstream layers

Layer architecture	Accuracy	Precision	Recall	F1-score
CLS token layer	*0.858 **(0.016)	0.823 (0.026)	0.835 (0.015)	0.827 (0.017)
**Two-token layer**	**0.867 (0.014)**	**0.840 (0.018)**	**0.847 (0.011)**	**0.842 (0.013)**
Three-token layer	0.861 (0.013)	0.835 (0.027)	0.842 (0.009)	0.836 (0.016)

The best scores are in bold

*Mean

**Standard deviation

The two-mask token layer showed better performance than the other two techniques. Even though it is not a large increase in performance, concatenating two vectors corresponding to entities and utilizing them for the prediction of relations is a better method than mapping the entire input sequence to only a [CLS] special token or even just using a [CLS] token, which is a representation of the entire sentence. Therefore, we can conclude that the two-token layer is the architecture that produces representations, which hold the most useful semantic information to predict a proper relation between the two entities. In other words, the number or location of the final output vectors in the downstream layer does matter in a RE task.

#### Final comparison of model performance

The experiments comparing different base models, input methodologies, and output layer structures using our datasets showed that utilizing PubMedBERT as a base model with the two masked sentence input methodology and two-token layer applied performs best. Finally, we compared this model with existing models presented in related works using our dataset. In addition to BERT-based models that have shown SOTA performance in relation extraction tasks, such as [36], we also included models based on other deep learning algorithms such as the CNN [38] and entity attention Bi-LSTM, which is a semantic relation classification model using bidirectional LSTM networks with entity-aware attention using latent entity typing [13]. In this final experiment, models were trained on a train set and evaluated on a validation set. The Table 9 reports the scores of the best models from the validation step evaluated on the test set.

**Table 9:** Performance comparison against models from related works

Model	F1
Word2vec + CNN [38]	0.708
Entity Attention Bi-LSTM [13]	0.787
Matching the Blanks [36]	0.799
**Our Model**	**0.852**

The best scores are in bold

**Fig. 8:**
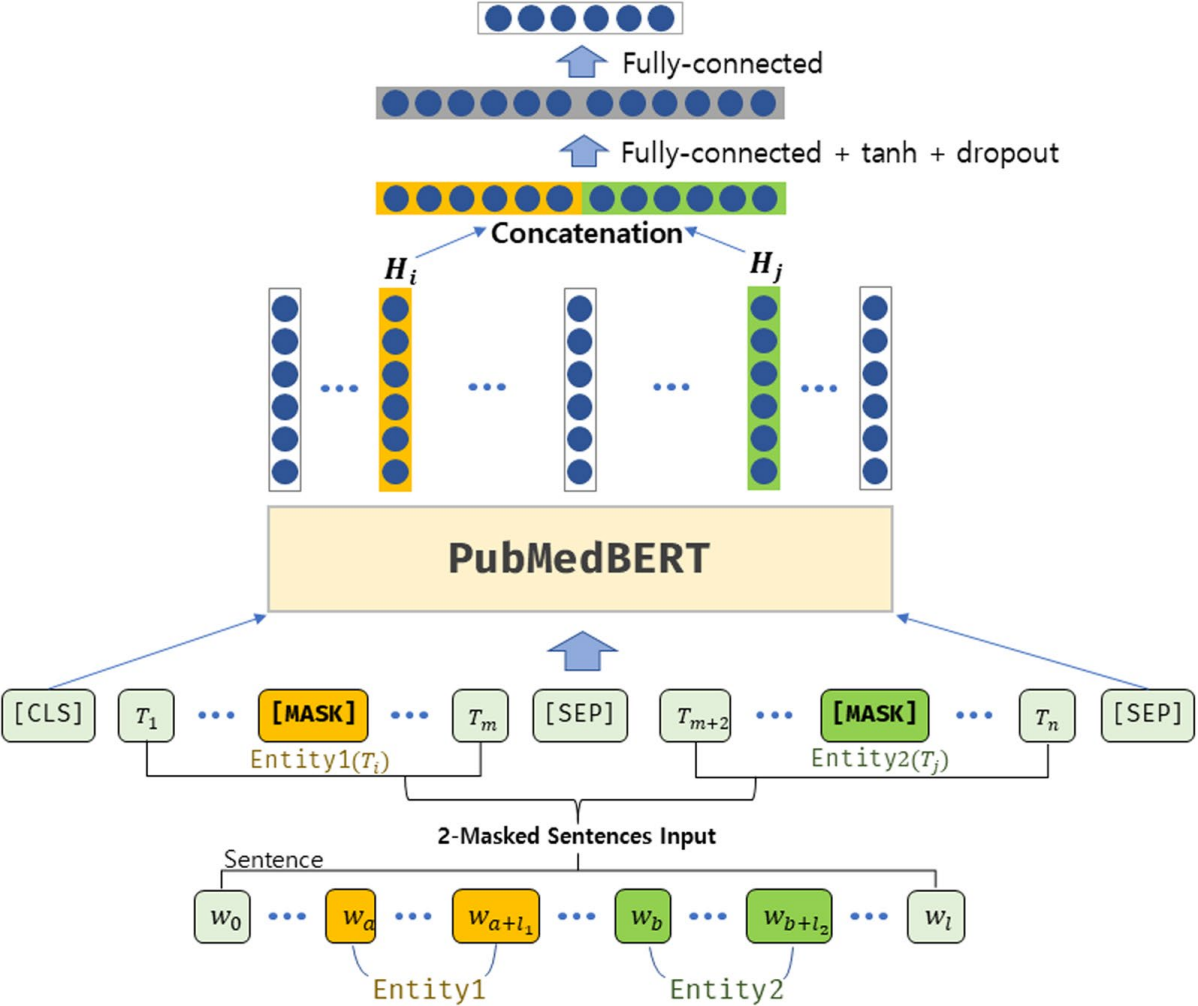
Best model: PubmedBERT-based model with the two masked sentence input method and two token layer applied

The experimental results confirm that the final proposed PubMedBERT-based model with the two masked sentence input methodology and two token layer performed best. The best model is illustrated in Fig. 8.

To determine how well our model predicts on each class and examine situations where our model has limitations, we further analyzed the per-class performance for each of the eight types of relations (Table 10).

**Table 10:** Per-class performance

Class	Precision	Recall	F1-score	Support
Directed Link	0.949	0.851	0.898	175
Negative Cause	0.902	0.888	0.895	156
Negative Decrease	0.909	0.918	0.914	74
Negative Increase	0.837	0.891	0.863	48
Positive Cause	0.907	0.895	0.901	206
Positive Decrease	0.714	0.741	0.727	26
Positive Increase	0.759	0.732	0.746	47
Undirected Link	0.840	0.906	0.872	275
			0.852	1007

Support: Number of instances in the test data represents 20% of the full data set, which is proportional to each class

In general, per-class performance relied on the number of data instances under the class. Classes designated as positive decrease, and positive increase, which had the fewest data instances of 26 and 47, respectively, obtained the lowest f1 scores among the different relation types. Other than the issue, the model showed generally even scores over the classes.

We closely examined the data points where the value predicted by the model differed from the annotated target value to objectively assess the limitations of our model or corpus and obtain insights for future reinforcement. As a result of the observation, we were able to identify two interesting patterns in false cases.

First, our model revealed its weakness when the verb between entities did not directly convey the meaning of increase/decrease or a cause-and-effect relationship, such as “improve,” “exacerbate,” and “aggravate,” making it difficult to accurately infer the relationship through context words surrounding entities. In this case, to correctly determine the direction in the quantity of the right entity, knowing whether the entity instance itself held a positive or negative meaning was necessary, such as in the sentence below:

Moreover, hepatic knockdown of HFREP1 improved insulin resistance in both mice fed a high-fat diet and ob/ob mice

The target relation type associating “HFREP1” and “insulin resistance” belongs to the negative decrease class, but the model incorrectly predicted it as the negative increase class. To accurately classify their relationship, in this case, the model needs to know whether insulin resistance itself has a positive or negative meaning. This type of error could be alleviated with a language model pre-trained on richer literature in the biomedical field, resulting in more comprehensive coverage of semantic meaning for bio-vocabulary.

Second, we found several cases of errors due to the conflict between the annotators’ contextual considerations of the entirety of the literature findings and the model predictions that exploit contextual words limited to each sentence in classifying the relationship between entities. An example of this is as follows:

Our data suggest that titanium particles may cause less leukocyte activation and inflammatory tissue responses than other particulate biomaterials used in total joint arthroplasty.

For this sentence, the annotator classified the relationship between titanium particles and inflammatory tissue response as the negative cause class, and the model predicted the positive cause class. The annotator compared the relationship between these two entities to other entities in the sentence and focused on the intention of the sentence. However, if we simply considered the directional association between the two entities of interest, we could assign the positive cause class, which was the model prediction.

To avoid this controversial gray area, the data that required abstract and complex consideration of context were excluded as much as possible from the corpus construction stage; as a result of this, few of these cases were found. However, we specifically paid attention to this example because it provided insights on how the model prediction works in these special circumstances and which direction to move forward in future research to overcome this limitation. In the example sentence, the model prediction cannot be regarded as wrong, but the main finding conveyed in the sentence must have been that titanium particles cause “less” inflammatory reactions, not the fact that they do. Therefore, this case demonstrates that meaningful relation types, which better reflect the intentions of the text and provide benefit to researchers, require elaborately reflecting not only the causality between entities and its direction but also the relative extent of the increased or decreased amount of a particular entity. This is possible by pushing beyond the limits of the current relation classification based on binary entities and addressing subtle and complicated interactions among multiple bio-entities appearing in a sentence.

## Conclusion

Machine learning-based relational classification tasks can be successfully performed based on good quality training data and well-designed algorithms. Especially, as Transformer-based algorithms become mainstream in NLP, the importance of quality datasets rather than complex feature engineering is increasing. Thus, constructing training datasets annotated with bio-entities and the relations between them is an urgent task to promote text mining research in biomedical fields.

In this paper, we developed a corpus with a wide range of bio-entities, such as biological processes, cells, compounds, DNA, enzymes, genes, hormones, molecular functions, phenotypes, proteins, RNA, and viruses, along with their annotated semantic relations. The construction of a corpus with multiple types of bio-entities and their rich relationships is essential to extracting complex and significant biological information from a wide range of bio-entity types that the biomedical literature contains. Considering this need, our newly constructed corpus, built by manually tagging a wide range of bio-entities and their relations from a rich amount of biomedical literature, represents a significant contribution. We comprehensively annotated verbs situated between entities, contextual information, such as positive/negative and active/passive information that affects their meanings, and other meaningful information in the sentence as features to consider in assigning semantic relation types, opening the possibility of further research on semantic relations. This corpus could be used as a reliable reference standard in the development of text mining systems.

Another contribution of this paper is that we demonstrated the utilization of the dataset that we built by training and evaluating BERT-based classification models leveraging the data and further presented a way to improve the performance of the relation classification task. Tweaking existing BERT-based models that are already known to show good performance for the classification task, we devised a new technique that can achieve better performance by alleviating the limitations of existing models for RE. By introducing a [MASK] token respectively on two identical input sentences, we effectively improved problems such as OOV words and inconsistency between pre-training and fine-tuning that afflict existing relation extraction models. In the overall comparison experiment between our model with all of the suggested methods applied and the existing models suggested in the related works, our proposed model showed the best performance, confirming that this methodology is effective in fine-tuning BERT-based pre-trained models for relation classification tasks.

In summary, the developed dataset for semantic relation classification was successfully applied to train the classification model. Therefore, this could be used as a valuable resource for similar text mining research. We also made significant improvements to the algorithms of the relation classification model. We expect that the biological information extracted with high accuracy through our proposed dataset and relation extraction technique will be used as a trusted source of information in the development of a biomedical text mining system. We also believe that the annotation processes we elaborated on here will be of significant help to fellow researchers performing similar work.

However, there remains room for improvement. To begin with, in the per-class performance analysis, our semantic relation based on the causality of binary entities and its direction showed limitations in sufficiently describing complex semantic associations among bio-entities in a sentence. For example, in cases where the relative intensity of the association needs to be revealed for meaningful knowledge discovery, the current relation type might be insufficient. To tackle this problem, introducing a complex semantic relation, of which the degree is higher than two, can be considered. Also, we are exploring ways of implementing RE with generative models such as T5, allowing the models to output sentences, which will be a direct and flexible answer reflecting the relation’s subtle nuance to the given prompt. Secondly, in the comparative experiment of the masking input method, while our proposed methods–two-sentence input, masked input, and combined or modified methods of these two–, were significantly superior to the original BERT method, the difference among them was trivial. Especially, our best method, two masked sentence input, outperformed entity marker-entity start, which was proposed in the previous study [36] by only a small margin. We additionally conducted an analysis of variance with the multiple measurements obtained through k-fold cross-validation, shown in Section D in Additional file 1, and the performance difference among non-original BERT methods was not statistically significant, although it was possible to determine which model or methodology is better than the rest. These minor differences between suggested structures warrant more in-depth future research, leading to a novel effective input treatment for RE providing significantly improved performance compared with the existing methods. If follow-up studies are conducted to address these listed limitations based on the realizations obtained through the experiments and analysis in this paper, we can expect further improvement in constructing a dataset and deep learning model for effective semantic relation classification to be achieved in the near future.

## Supplementary Information

**Additional file 1**. Overview of Corpora.

## Data Availability

The datasets generated during the current study are available at https://github.com/tsmmbio/BertSRC*.*

## Footnotes

1 Text annotation editor. TextAE, 2020. Accessed 2021 Aug 30. Available from: https://textae.pubannotation.org/
