# Supplementary material for: BertSRC: transformer-based semantic relation classification
Source: BMC Med Inform Decis Mak. 2022 Sep 6;22:234. doi: 10.1186/s12911-022-01977-5 (PMC9446816; doi:10.1186/s12911-022-01977-5)
Supplement: Supplementary file 1 — Additional file 1. Overview of Corpora. [file 12911_2022_1977_MOESM1_ESM.docx]

1. **Overview of Corpora**

**A-1. Benchmark corpora Comparison**

|  | Description | Size | Entity | Relation | Negative Example | Hierarchy of Relation | Direction of Relation | Text Binding for Relation | Text Unit |
| --- | --- | --- | --- | --- | --- | --- | --- | --- | --- |
| GENIA[49, 50] | REL task dataset of the BioNLP Shared Task 2011  Retrieved with Mesh terms "human", "blood cells", "transcription factors"  annotate linguistic and semantic information | 1999 articles | 2 | 2 | YES | x | YES | x | Sentence |
|  |  |  | Protein (gene, or gene product)  Entity (non-protein) | Subunit-Complex (arg1: Protein, arg2: Entity)  Protein-Component (arg1: Protein, arg2: Entity) |  |  | Protein types → Entity types |  |  |
| LLL05  [51, 52] | Annotate gene interaction in a model of bacterium Bacillus subtilis  Agent - target pair interaction  Interaction is defined:  1) Explicit action  2) Binding of a protein on the promoter of the target gene  3) Membership in a regulon family | 106 instances without coreferences  165 instances with coreferences  80 sentences | 2 | x | x | x | YES | x | Sentence |
|  |  |  | Agent (protein)  Target (gene) |  |  |  | Agent → Target |  |  |
| BioCreative-II  [53] | Dataset from Task 2, Protein Interaction Pairs Sub-task 2 | 4637 instances  1098 articles | x | x | x | x | x | x | Full text |
|  |  |  | Protein |  |  |  |  |  |  |
| AIMed[54] |  | 225 Articles  1101 instances  1955 sentences | 11 | x | YES | YES | x | x | Sentence |
|  |  |  | A list of gene/protein names |  |  |  |  |  |  |
| BioInfer [55] | Entities, relations, and dependencies are annotated for each sentence.  Based on ontology with complex relation structure but be decomposed into a binary relation | 1100 sentences | 70+ | 60+ | YES | YES | YES | YES | Sentence |
|  |  |  | In addition to named entities for physical entities, such as proteins, genes, and RNA, process attributes, such as location, interaction, effect, and amount, representing ontology, are regarded as entities and become an argument of the relation. | The most general four classes-observation, part_of, is_a, causal- and branched out granular classes (a group of pre-defined relation types + heuristically discovered predicates)  The structure of relation ontology is stratified into a maximum of five levels, and the identified relationship between entities is classified as the most detailed type |  |  |  |  |  |
| HPRD50 [56] | Automatically annotate entities using NER software ProMiner and manually annotate relation between them  Interaction is defined as:  1. Direct physical interactions  2. Regulatory relation physical modification | 50 articles  432 instances  145 sentences | x | x | YES | x | x | x | Sentence |
|  |  |  | Gene/protein |  |  |  |  |  |  |
| IEPA [57] | Annotate from PubMed abstracts containing co-occurring chemical pairs  Interaction is a "direct or indirect influence of one on the quantity or activity of the other". | 486 sentences | x | x | YES | x | YES | YES | Phrase  sentence  abstract |
|  |  |  | List of 10 pairs of chemicals (16 distinct terms) |  |  |  |  |  |  |

Size: Size of the corpus. Each corpus has a different structure and format; thus, it was improbable to standardize size units (article, sentence, relation instance). In the case of instances, the number of negative examples is included.

Entity: Entity type and number. If the type is not defined separately, it is expressed as ‘x.’

Relation: Relation type and number. If the type is not defined separately, it is expressed as ‘x.’

Negative Examples: Whether annotations of negative examples are included. If not, it is denoted as ‘x’.

Hierarchy of relation: Whether there is a hierarchical distinction or a nested relationship between relation types.

Direction of relation: Whether there is a direction of the relationship between the two entities.

Text binding: Whether a specific part of the text that was a clue to determining the relation has been identified.

Text unit: A unit from which a relation is annotated.

**A-2. Corpus presented in this work**

| **Our Corpus** | SIZE | ENTITY | RELATION | NEGATIVE EXAMPLES | HIERARCHY | DIRECTION | TEXT BINDING | UNIT |
| --- | --- | --- | --- | --- | --- | --- | --- | --- |
|  | 1,346 abstracts  5,045 relation instances | 12 | 8 | Yes | Yes | Yes | Yes | sentence |
|  |  | biological processes  cells  compounds  DNA  enzymes  genes  hormones  molecular functions  phenotypes  proteins  RNA  viruses | Undirected Link  Directed Link  Positive Cause  Positive Increase  Negative Decrease  Negative Cause  Positive Decrease  Negative Increase | Undirected Link | For reflecting certainty | Except for Undirected Link, which is a negative relation, seven relation types are defined that are highly based on the amount of directionality for each entity. | Context words around entities and verbs are annotated along with entities and relations. |  |

1. **Entity types**

**Biological Process**

A biological process is a process or an action that occurs within a cell. Biological processes include pathways, processes, cascades, metabolism, cycles, and phases. Chemical reactions, such as phosphorylation, methylation, aminoacylation, acetylation, and nitrosylation, and the transport, secretion, exchange, export, and import of ions, amino acids (AAs), proteins, compounds, and molecular formulas are also biological processes. DNA replication, DNA damage, DNA transcription, translation, excision, RNA splicing, and DNA elongation are also biological processes.

**Cell, Virus**

Experiments are conducted on several species. Cell and virus are annotated within the sentence when an experiment is performed on a cell or virus or when a specific cell or virus name is present.

**Compound**

Compounds include additives such as drugs and chemicals and *in vivo* products, such as amino acids, lipids, and proteins. However, if the entity binds to other proteins or substances in the body and affects them, it is classified as a molecular function.

**Gene, DNA, RNA, Protein**

In biology, a gene is the functional unit of heredity and the nucleotide sequence of DNA or RNA that holds instructions for the synthesis of either RNA or protein [41-43]. In cellular organisms, protein generation according to the information encoded in the DNA of a gene involves two steps: transcription and translation [44]. It is not easy to distinguish between genes and proteins because they often have the same name. Different species have different methods of labeling genes and proteins; thus, manually reviewing the annotation is necessary.

**Hormone**

A hormone is any member of a class of signaling molecules in multicellular organisms that is transported to organs and tissues in the body to regulate physiology and behavior [45]. Hormones enable communication between organs and tissues. In vertebrates, hormones are responsible for the regulation of many physiological processes and behavioral activities, such as digestion, metabolism, respiration, sensory perception, sleep, excretion, lactation, stress induction, growth and development, movement, reproduction, and mood manipulation [46, 47]. In plants, hormones modulate almost all aspects of development from germination to senescence [48].

**Molecular Function**

Proteins that have a binding role, such as hormones and antigen-antibodies, are called molecular functions (e.g., antigens, antibodies, serum proteins, major histocompatibility complexes (MHCs), and signal transduction materials such as lipoproteins, SMADs, and toxins).

**Phenotype**

The original dictionary definition of phenotype is the observable characteristics of an organism due to genetic and environmental influences. However, we annotate phenotype by limiting it to disease. Symptoms, injuries, diseases, syndromes, and illnesses belong to phenotypes. However, since cell death is an intracellular activity, it is annotated as a biological process rather than a phenotype. In the case of tumors and cancer cells, they are classified and annotated as phenotypes since they are related to diseases.

1. **Examples for Each Relation Type**

Bold text indicates means an entity, [] indicates context words, and ‘_’ indicates a relation trigger verb.

| **Relation type** | **Sentence** | **Reference (PMID)** |
| --- | --- | --- |
| **Directed Link**  **-**DL (*HAI-2*, *prostasin)*  -DL (*HAI-2*, *matriptase*) | ***HAI-2*** *has been implicated in the regulation of the activities of several serine proteases including* ***prostasin*** *and* ***matriptase****, which are both important for epithelial barrier formation.* | #30445423, Sentence #6 |
|  | Explanation:  The verb "regulate" expresses a direct effect; thus, it was determined that there was a causal relationship. However, since the direction of causality was not explicitly revealed in this sentence, the entity was classified as a directed link without being classified to a more detailed stage. |  |
| **Positive Cause**  **-**PC (*ER stress response*, *apoptosis)*  -PC (*ER stress response*, *oxidative stress)* | *These findings indicate that recurrent short-term hypoglycemia and hyperglycemia induce* ***apoptosis*** *and* ***oxidative stress*** *via the* ***ER stress response*** *in Schwann cells.* | #30444976, Sentence #9 |
|  | Explanation:  The verb "induce" indicates a positive causal relation, meaning “to make something happen, exist, or increase in amount”. Here, since no expression about the amount changed was specified, the relation was not subdivided into the positive increase/negative decrease classes, both of which are subcategories of the positive cause class because the degree of change in their arguments is in the same direction (positive(+) increase(+), negative(-) decrease(-)). |  |
| **Positive Increase**  **-**PI (*GPR40, GSIS*) | *Inhibitors of phospholipase C or protein kinase C PKC inhibited the [increases in]* ***GSIS*** *and the NSCC current induced by* ***GPR40*** *[stimulation].* | #27180622 , Sentence #7 |
|  | Explanation:  Although this sentence specifies an interaction mediated by the same verb "induce" (as in #30444976 Sentence #9), we annotated this relation as a positive increase here because explicit context words denoting an increase in amount (increases in, stimulation) were located next to each entity. |  |
| **Negative Decrease**  **-**ND(*HOTTIP, Akt1*) | *Additionally, HOTTIP [knockdown] downregulated Akt1 expression and suppressed cell proliferation, invasion and migration in PTC cells by regulating miR-637.* | #29474928, Sentence #11 |
|  | Explanation:  Since 'knockdown' of HOTTIP reduced the amount of Akt1, these two entities are in a positive causal relationship, the negative decrease class was assigned because quantitative expressions, such as knockdown and downregulated, were present. |  |
| **Negative Cause**  -NC(*PEPCK1 ASO, white adipose tissue mass)* | *In contrast,* ***PEPCK1 ASO*** *decreased the* ***white adipose tissue mass*** *in HFF rats but without altering basal rates of lipolysis, de novo lipogenesis, or glyceroneogenesis in vivo.* | #30445425, Sentence #5 |
|  | Explanation:  This relation was annotated to the negative cause class because there were no explicit quantitative context words. |  |
| **Positive Decrease**  -PD (*glucose*, *cirHIPK3*) | *[High]* ***glucose****-induced* ***circHIPK3*** *[downregulation] mediates endothelial cell injury.* | #30454897, Sentence #0 |
|  | Explanation:  This relation was considered a positive decrease because a negative causal relationship and explicit quantitative expression ('high,' 'downregulation') specifying changes in the amount of each entity were present. |  |
| **Negative Increase**  -NI (*DCLK1-B, apoptosis)* | ***DCLK1-B*** *[depletion] impairs cancer stemness resulting in reduced survival potential and increased* ***apoptosis****, thus sensitizing colorectal cancer to chemoradiation.* | #30446587, Sentence #9 |
|  | Explanation:  There is a negative causal relationship, and the context word "depletion" for the 'DCLK1-B' entity and trigger verb, "increase," specify quantitative changes; thus, this was annotated as a negative increase. |  |
| **Undirected Link** | *In addition,* ***MoAbp1*** *interacts with* ***MoCap*** *(adenylyl cyclase-associated protein) affecting its normal patch localization pattern and the actin protein MoAct1 through its conserved domains.* | #30451565, Sentence #6 |
|  | Explanation:  A pair of entities linked by verbs, such as "interact," "associate," and "correlate," or the conjunction "and" in parallel were mostly annotated as an undirected link. |  |

1. **One-way ANOVA analysis**

**D-1. Performance comparison of pre-trained language models**

| **ANOVA** | | | | | | |
| --- | --- | --- | --- | --- | --- | --- |
|  | | Sum of Squares | df | Mean Square | F | Sig. |
| accuracy | Between Groups | .001 | 4 | .000 | 2.411 | .083 |
|  | Within Groups | .002 | 20 | .000 |  |  |
|  | Total | .003 | 24 |  |  |  |
| precision | Between Groups | .001 | 4 | .000 | 1.166 | .356 |
|  | Within Groups | .006 | 20 | .000 |  |  |
|  | Total | .007 | 24 |  |  |  |
| recall | Between Groups | .002 | 4 | .001 | 3.238 | .033 |
|  | Within Groups | .004 | 20 | .000 |  |  |
|  | Total | .006 | 24 |  |  |  |
| fscore | Between Groups | .002 | 4 | .000 | 2.845 | .051 |
|  | Within Groups | .003 | 20 | .000 |  |  |
|  | Total | .005 | 24 |  |  |  |

| **ANOVA Effect Sizes**^a,b^ | | | | |
| --- | --- | --- | --- | --- |
|  | | Point Estimate | 95% Confidence Interval | |
|  |  |  | Lower | Upper |
| accuracy | Eta-squared | .325 | .000 | .489 |
|  | Epsilon-squared | .190 | -.200 | .386 |
|  | Omega-squared Fixed-effect | .184 | -.190 | .377 |
|  | Omega-squared Random-effect | .053 | -.042 | .131 |
| precision | Eta-squared | .189 | .000 | .352 |
|  | Epsilon-squared | .027 | -.200 | .223 |
|  | Omega-squared Fixed-effect | .026 | -.190 | .216 |
|  | Omega-squared Random-effect | .007 | -.042 | .064 |
| recall | Eta-squared | .393 | .000 | .547 |
|  | Epsilon-squared | .272 | -.200 | .457 |
|  | Omega-squared Fixed-effect | .264 | -.190 | .447 |
|  | Omega-squared Random-effect | .082 | -.042 | .168 |
| fscore | Eta-squared | .363 | .000 | .522 |
|  | Epsilon-squared | .235 | -.200 | .426 |
|  | Omega-squared Fixed-effect | .228 | -.190 | .416 |
|  | Omega-squared Random-effect | .069 | -.042 | .151 |
| a. Eta-squared and Epsilon-squared are estimated based on the fixed-effect model. | | | | |
| b. Negative but less biased estimates are retained, not rounded to zero. | | | | |

**Post Hoc Tests**

| **Multiple Comparisons** | | | | | | | |
| --- | --- | --- | --- | --- | --- | --- | --- |
| Tukey HSD | | | | | | | |
| Dependent Variable | (I) model | (J) model | Mean Difference (I-J) | Std. Error | Sig. | 95% Confidence Interval | |
|  |  |  |  |  |  | Lower Bound | Upper Bound |
| accuracy | bert | biobert | -.01269 | .00593 | .243 | -.0304 | .0051 |
|  |  | pubmedbert | -.01665 | .00593 | .073 | -.0344 | .0011 |
|  |  | roberta | -.01388 | .00593 | .174 | -.0316 | .0039 |
|  |  | scibert | -.01348 | .00593 | .195 | -.0312 | .0043 |
|  | biobert | bert | .01269 | .00593 | .243 | -.0051 | .0304 |
|  |  | pubmedbert | -.00396 | .00593 | .961 | -.0217 | .0138 |
|  |  | roberta | -.00119 | .00593 | 1.000 | -.0189 | .0166 |
|  |  | scibert | -.00079 | .00593 | 1.000 | -.0185 | .0170 |
|  | pubmedbert | bert | .01665 | .00593 | .073 | -.0011 | .0344 |
|  |  | biobert | .00396 | .00593 | .961 | -.0138 | .0217 |
|  |  | roberta | .00278 | .00593 | .989 | -.0150 | .0205 |
|  |  | scibert | .00317 | .00593 | .983 | -.0146 | .0209 |
|  | roberta | bert | .01388 | .00593 | .174 | -.0039 | .0316 |
|  |  | biobert | .00119 | .00593 | 1.000 | -.0166 | .0189 |
|  |  | pubmedbert | -.00278 | .00593 | .989 | -.0205 | .0150 |
|  |  | scibert | .00040 | .00593 | 1.000 | -.0174 | .0181 |
|  | scibert | bert | .01348 | .00593 | .195 | -.0043 | .0312 |
|  |  | biobert | .00079 | .00593 | 1.000 | -.0170 | .0185 |
|  |  | pubmedbert | -.00317 | .00593 | .983 | -.0209 | .0146 |
|  |  | roberta | -.00040 | .00593 | 1.000 | -.0181 | .0174 |
| precision | bert | biobert | -.01779 | .01058 | .467 | -.0495 | .0139 |
|  |  | pubmedbert | -.01610 | .01058 | .561 | -.0478 | .0156 |
|  |  | roberta | -.01813 | .01058 | .449 | -.0498 | .0135 |
|  |  | scibert | -.01945 | .01058 | .381 | -.0511 | .0122 |
|  | biobert | bert | .01779 | .01058 | .467 | -.0139 | .0495 |
|  |  | pubmedbert | .00170 | .01058 | 1.000 | -.0300 | .0334 |
|  |  | roberta | -.00034 | .01058 | 1.000 | -.0320 | .0313 |
|  |  | scibert | -.00166 | .01058 | 1.000 | -.0333 | .0300 |
|  | pubmedbert | bert | .01610 | .01058 | .561 | -.0156 | .0478 |
|  |  | biobert | -.00170 | .01058 | 1.000 | -.0334 | .0300 |
|  |  | roberta | -.00203 | .01058 | 1.000 | -.0337 | .0296 |
|  |  | scibert | -.00335 | .01058 | .998 | -.0350 | .0283 |
|  | roberta | bert | .01813 | .01058 | .449 | -.0135 | .0498 |
|  |  | biobert | .00034 | .01058 | 1.000 | -.0313 | .0320 |
|  |  | pubmedbert | .00203 | .01058 | 1.000 | -.0296 | .0337 |
|  |  | scibert | -.00132 | .01058 | 1.000 | -.0330 | .0303 |
|  | scibert | bert | .01945 | .01058 | .381 | -.0122 | .0511 |
|  |  | biobert | .00166 | .01058 | 1.000 | -.0300 | .0333 |
|  |  | pubmedbert | .00335 | .01058 | .998 | -.0283 | .0350 |
|  |  | roberta | .00132 | .01058 | 1.000 | -.0303 | .0330 |
| recall | bert | biobert | -.02406 | .00839 | .064 | -.0492 | .0011 |
|  |  | pubmedbert | -.02699^*^ | .00839 | .031 | -.0521 | -.0019 |
|  |  | roberta | -.01502 | .00839 | .406 | -.0401 | .0101 |
|  |  | scibert | -.02078 | .00839 | .136 | -.0459 | .0043 |
|  | biobert | bert | .02406 | .00839 | .064 | -.0011 | .0492 |
|  |  | pubmedbert | -.00293 | .00839 | .997 | -.0280 | .0222 |
|  |  | roberta | .00904 | .00839 | .816 | -.0161 | .0341 |
|  |  | scibert | .00328 | .00839 | .995 | -.0218 | .0284 |
|  | pubmedbert | bert | .02699^*^ | .00839 | .031 | .0019 | .0521 |
|  |  | biobert | .00293 | .00839 | .997 | -.0222 | .0280 |
|  |  | roberta | .01196 | .00839 | .619 | -.0131 | .0371 |
|  |  | scibert | .00621 | .00839 | .944 | -.0189 | .0313 |
|  | roberta | bert | .01502 | .00839 | .406 | -.0101 | .0401 |
|  |  | biobert | -.00904 | .00839 | .816 | -.0341 | .0161 |
|  |  | pubmedbert | -.01196 | .00839 | .619 | -.0371 | .0131 |
|  |  | scibert | -.00576 | .00839 | .957 | -.0309 | .0194 |
|  | scibert | bert | .02078 | .00839 | .136 | -.0043 | .0459 |
|  |  | biobert | -.00328 | .00839 | .995 | -.0284 | .0218 |
|  |  | pubmedbert | -.00621 | .00839 | .944 | -.0313 | .0189 |
|  |  | roberta | .00576 | .00839 | .957 | -.0194 | .0309 |
| fscore | bert | biobert | -.02092 | .00763 | .083 | -.0438 | .0019 |
|  |  | pubmedbert | -.02161 | .00763 | .069 | -.0444 | .0012 |
|  |  | roberta | -.01684 | .00763 | .218 | -.0397 | .0060 |
|  |  | scibert | -.02036 | .00763 | .095 | -.0432 | .0025 |
|  | biobert | bert | .02092 | .00763 | .083 | -.0019 | .0438 |
|  |  | pubmedbert | -.00069 | .00763 | 1.000 | -.0235 | .0221 |
|  |  | roberta | .00408 | .00763 | .983 | -.0188 | .0269 |
|  |  | scibert | .00056 | .00763 | 1.000 | -.0223 | .0234 |
|  | pubmedbert | bert | .02161 | .00763 | .069 | -.0012 | .0444 |
|  |  | biobert | .00069 | .00763 | 1.000 | -.0221 | .0235 |
|  |  | roberta | .00477 | .00763 | .969 | -.0181 | .0276 |
|  |  | scibert | .00125 | .00763 | 1.000 | -.0216 | .0241 |
|  | roberta | bert | .01684 | .00763 | .218 | -.0060 | .0397 |
|  |  | biobert | -.00408 | .00763 | .983 | -.0269 | .0188 |
|  |  | pubmedbert | -.00477 | .00763 | .969 | -.0276 | .0181 |
|  |  | scibert | -.00352 | .00763 | .990 | -.0263 | .0193 |
|  | scibert | bert | .02036 | .00763 | .095 | -.0025 | .0432 |
|  |  | biobert | -.00056 | .00763 | 1.000 | -.0234 | .0223 |
|  |  | pubmedbert | -.00125 | .00763 | 1.000 | -.0241 | .0216 |
|  |  | roberta | .00352 | .00763 | .990 | -.0193 | .0263 |
| *. The mean difference is significant at the 0.05 level. | | | | | | | |

**Homogeneous Subsets**

| **accuracy** | | |
| --- | --- | --- |
| Tukey HSD^a^ | | |
| model | N | Subset for alpha = 0.05 |
|  |  | 1 |
| bert | 5 | .8486 |
| biobert | 5 | .8612 |
| scibert | 5 | .8620 |
| roberta | 5 | .8624 |
| pubmedbert | 5 | .8652 |
| Sig. |  | .073 |
| Means for groups in homogeneous subsets are displayed. | | |
| a. Uses Harmonic Mean Sample Size = 5.000. | | |

| **precision** | | |
| --- | --- | --- |
| Tukey HSD^a^ | | |
| model | N | Subset for alpha = 0.05 |
|  |  | 1 |
| bert | 5 | .8167 |
| pubmedbert | 5 | .8328 |
| biobert | 5 | .8345 |
| roberta | 5 | .8348 |
| scibert | 5 | .8361 |
| Sig. |  | .381 |
| Means for groups in homogeneous subsets are displayed. | | |
| a. Uses Harmonic Mean Sample Size = 5.000. | | |

| **recall** | | | |
| --- | --- | --- | --- |
| Tukey HSD^a^ | | | |
| model | N | Subset for alpha = 0.05 | |
|  |  | 1 | 2 |
| bert | 5 | .8223 |  |
| roberta | 5 | .8373 | .8373 |
| scibert | 5 | .8431 | .8431 |
| biobert | 5 | .8464 | .8464 |
| pubmedbert | 5 |  | .8493 |
| Sig. |  | .064 | .619 |
| Means for groups in homogeneous subsets are displayed. | | | |
| a. Uses Harmonic Mean Sample Size = 5.000. | | | |

| **fscore** | | |
| --- | --- | --- |
| Tukey HSD^a^ | | |
| model | N | Subset for alpha = 0.05 |
|  |  | 1 |
| bert | 5 | .8177 |
| roberta | 5 | .8346 |
| scibert | 5 | .8381 |
| biobert | 5 | .8387 |
| pubmedbert | 5 | .8393 |
| Sig. |  | .069 |
| Means for groups in homogeneous subsets are displayed. | | |
| a. Uses Harmonic Mean Sample Size = 5.000. | | |

**D-2. Performance comparison of masking input methods**

| **ANOVA** | | | | | | |
| --- | --- | --- | --- | --- | --- | --- |
|  | | Sum of Squares | df | Mean Square | F | Sig. |
| accuracy | Between Groups | .102 | 4 | .025 | 129.411 | <.001 |
|  | Within Groups | .004 | 20 | .000 |  |  |
|  | Total | .106 | 24 |  |  |  |
| precision | Between Groups | .135 | 4 | .034 | 71.318 | <.001 |
|  | Within Groups | .009 | 20 | .000 |  |  |
|  | Total | .144 | 24 |  |  |  |
| recall | Between Groups | .147 | 4 | .037 | 236.314 | <.001 |
|  | Within Groups | .003 | 20 | .000 |  |  |
|  | Total | .150 | 24 |  |  |  |
| fscore | Between Groups | .145 | 4 | .036 | 182.398 | <.001 |
|  | Within Groups | .004 | 20 | .000 |  |  |
|  | Total | .149 | 24 |  |  |  |

| **ANOVA Effect Sizes**^a^ | | | | |
| --- | --- | --- | --- | --- |
|  | | Point Estimate | 95% Confidence Interval | |
|  |  |  | Lower | Upper |
| accuracy | Eta-squared | .963 | .904 | .973 |
|  | Epsilon-squared | .955 | .885 | .967 |
|  | Omega-squared Fixed-effect | .954 | .881 | .966 |
|  | Omega-squared Random-effect | .837 | .649 | .877 |
| precision | Eta-squared | .934 | .833 | .952 |
|  | Epsilon-squared | .921 | .800 | .943 |
|  | Omega-squared Fixed-effect | .918 | .793 | .941 |
|  | Omega-squared Random-effect | .738 | .490 | .798 |
| recall | Eta-squared | .979 | .946 | .985 |
|  | Epsilon-squared | .975 | .936 | .982 |
|  | Omega-squared Fixed-effect | .974 | .933 | .981 |
|  | Omega-squared Random-effect | .904 | .777 | .928 |
| fscore | Eta-squared | .973 | .931 | .981 |
|  | Epsilon-squared | .968 | .917 | .977 |
|  | Omega-squared Fixed-effect | .967 | .914 | .976 |
|  | Omega-squared Random-effect | .879 | .727 | .909 |
| a. Eta-squared and Epsilon-squared are estimated based on the fixed-effect model. | | | | |

**Post Hoc Tests**

| **Multiple Comparisons** | | | | | | | |
| --- | --- | --- | --- | --- | --- | --- | --- |
| Tukey HSD | | | | | | | |
| Dependent Variable | (I) method | (J) method | Mean Difference (I-J) | Std. Error | Sig. | 95% Confidence Interval | |
|  |  |  |  |  |  | Lower Bound | Upper Bound |
| accuracy | 2-masked-sentence | 2sentence-entity-token | .00139 | .00886 | 1.000 | -.0251 | .0279 |
|  |  | default | .16630^*^ | .00886 | <.001 | .1398 | .1928 |
|  |  | entity marker entity start | .00912 | .00886 | .839 | -.0174 | .0356 |
|  |  | masked | .02180 | .00886 | .140 | -.0047 | .0483 |
|  | 2sentence-entity-token | 2-masked-sentence | -.00139 | .00886 | 1.000 | -.0279 | .0251 |
|  |  | default | .16492^*^ | .00886 | <.001 | .1384 | .1914 |
|  |  | entity marker entity start | .00773 | .00886 | .904 | -.0188 | .0342 |
|  |  | masked | .02042 | .00886 | .185 | -.0061 | .0469 |
|  | default | 2-masked-sentence | -.16630^*^ | .00886 | <.001 | -.1928 | -.1398 |
|  |  | 2sentence-entity-token | -.16492^*^ | .00886 | <.001 | -.1914 | -.1384 |
|  |  | entity marker entity start | -.15719^*^ | .00886 | <.001 | -.1837 | -.1307 |
|  |  | masked | -.14450^*^ | .00886 | <.001 | -.1710 | -.1180 |
|  | entity marker entity start | 2-masked-sentence | -.00912 | .00886 | .839 | -.0356 | .0174 |
|  |  | 2sentence-entity-token | -.00773 | .00886 | .904 | -.0342 | .0188 |
|  |  | default | .15719^*^ | .00886 | <.001 | .1307 | .1837 |
|  |  | masked | .01269 | .00886 | .616 | -.0138 | .0392 |
|  | masked | 2-masked-sentence | -.02180 | .00886 | .140 | -.0483 | .0047 |
|  |  | 2sentence-entity-token | -.02042 | .00886 | .185 | -.0469 | .0061 |
|  |  | default | .14450^*^ | .00886 | <.001 | .1180 | .1710 |
|  |  | entity marker entity start | -.01269 | .00886 | .616 | -.0392 | .0138 |
| precision | 2-masked-sentence | 2sentence-entity-token | -.00232 | .01376 | 1.000 | -.0435 | .0389 |
|  |  | default | .19075^*^ | .01376 | <.001 | .1496 | .2319 |
|  |  | entity marker entity start | .01167 | .01376 | .912 | -.0295 | .0528 |
|  |  | masked | .02539 | .01376 | .377 | -.0158 | .0666 |
|  | 2sentence-entity-token | 2-masked-sentence | .00232 | .01376 | 1.000 | -.0389 | .0435 |
|  |  | default | .19307^*^ | .01376 | <.001 | .1519 | .2342 |
|  |  | entity marker entity start | .01399 | .01376 | .845 | -.0272 | .0552 |
|  |  | masked | .02771 | .01376 | .295 | -.0135 | .0689 |
|  | default | 2-masked-sentence | -.19075^*^ | .01376 | <.001 | -.2319 | -.1496 |
|  |  | 2sentence-entity-token | -.19307^*^ | .01376 | <.001 | -.2342 | -.1519 |
|  |  | entity marker entity start | -.17908^*^ | .01376 | <.001 | -.2203 | -.1379 |
|  |  | masked | -.16536^*^ | .01376 | <.001 | -.2065 | -.1242 |
|  | entity marker entity start | 2-masked-sentence | -.01167 | .01376 | .912 | -.0528 | .0295 |
|  |  | 2sentence-entity-token | -.01399 | .01376 | .845 | -.0552 | .0272 |
|  |  | default | .17908^*^ | .01376 | <.001 | .1379 | .2203 |
|  |  | masked | .01372 | .01376 | .854 | -.0275 | .0549 |
|  | masked | 2-masked-sentence | -.02539 | .01376 | .377 | -.0666 | .0158 |
|  |  | 2sentence-entity-token | -.02771 | .01376 | .295 | -.0689 | .0135 |
|  |  | default | .16536^*^ | .01376 | <.001 | .1242 | .2065 |
|  |  | entity marker entity start | -.01372 | .01376 | .854 | -.0549 | .0275 |
| recall | 2-masked-sentence | 2sentence-entity-token | .00194 | .00789 | .999 | -.0217 | .0255 |
|  |  | default | .19926^*^ | .00789 | <.001 | .1757 | .2229 |
|  |  | entity marker entity start | .01083 | .00789 | .651 | -.0128 | .0344 |
|  |  | masked | .02075 | .00789 | .102 | -.0029 | .0444 |
|  | 2sentence-entity-token | 2-masked-sentence | -.00194 | .00789 | .999 | -.0255 | .0217 |
|  |  | default | .19732^*^ | .00789 | <.001 | .1737 | .2209 |
|  |  | entity marker entity start | .00889 | .00789 | .791 | -.0147 | .0325 |
|  |  | masked | .01881 | .00789 | .161 | -.0048 | .0424 |
|  | default | 2-masked-sentence | -.19926^*^ | .00789 | <.001 | -.2229 | -.1757 |
|  |  | 2sentence-entity-token | -.19732^*^ | .00789 | <.001 | -.2209 | -.1737 |
|  |  | entity marker entity start | -.18843^*^ | .00789 | <.001 | -.2120 | -.1648 |
|  |  | masked | -.17852^*^ | .00789 | <.001 | -.2021 | -.1549 |
|  | entity marker entity start | 2-masked-sentence | -.01083 | .00789 | .651 | -.0344 | .0128 |
|  |  | 2sentence-entity-token | -.00889 | .00789 | .791 | -.0325 | .0147 |
|  |  | default | .18843^*^ | .00789 | <.001 | .1648 | .2120 |
|  |  | masked | .00992 | .00789 | .719 | -.0137 | .0335 |
|  | masked | 2-masked-sentence | -.02075 | .00789 | .102 | -.0444 | .0029 |
|  |  | 2sentence-entity-token | -.01881 | .00789 | .161 | -.0424 | .0048 |
|  |  | default | .17852^*^ | .00789 | <.001 | .1549 | .2021 |
|  |  | entity marker entity start | -.00992 | .00789 | .719 | -.0335 | .0137 |
| fscore | 2-masked-sentence | 2sentence-entity-token | .00039 | .00891 | 1.000 | -.0263 | .0271 |
|  |  | default | .19805^*^ | .00891 | <.001 | .1714 | .2247 |
|  |  | entity marker entity start | .01184 | .00891 | .678 | -.0148 | .0385 |
|  |  | masked | .02350 | .00891 | .101 | -.0032 | .0502 |
|  | 2sentence-entity-token | 2-masked-sentence | -.00039 | .00891 | 1.000 | -.0271 | .0263 |
|  |  | default | .19767^*^ | .00891 | <.001 | .1710 | .2243 |
|  |  | entity marker entity start | .01145 | .00891 | .703 | -.0152 | .0381 |
|  |  | masked | .02311 | .00891 | .110 | -.0036 | .0498 |
|  | default | 2-masked-sentence | -.19805^*^ | .00891 | <.001 | -.2247 | -.1714 |
|  |  | 2sentence-entity-token | -.19767^*^ | .00891 | <.001 | -.2243 | -.1710 |
|  |  | entity marker entity start | -.18621^*^ | .00891 | <.001 | -.2129 | -.1595 |
|  |  | masked | -.17455^*^ | .00891 | <.001 | -.2012 | -.1479 |
|  | entity marker entity start | 2-masked-sentence | -.01184 | .00891 | .678 | -.0385 | .0148 |
|  |  | 2sentence-entity-token | -.01145 | .00891 | .703 | -.0381 | .0152 |
|  |  | default | .18621^*^ | .00891 | <.001 | .1595 | .2129 |
|  |  | masked | .01166 | .00891 | .689 | -.0150 | .0383 |
|  | masked | 2-masked-sentence | -.02350 | .00891 | .101 | -.0502 | .0032 |
|  |  | 2sentence-entity-token | -.02311 | .00891 | .110 | -.0498 | .0036 |
|  |  | default | .17455^*^ | .00891 | <.001 | .1479 | .2012 |
|  |  | entity marker entity start | -.01166 | .00891 | .689 | -.0383 | .0150 |
| *. The mean difference is significant at the 0.05 level. | | | | | | | |

**Homogeneous Subsets**

| **accuracy** | | | |
| --- | --- | --- | --- |
| Tukey HSD^a^ | | | |
| method | N | Subset for alpha = 0.05 | |
|  |  | 1 | 2 |
| default | 5 | .6997 |  |
| masked | 5 |  | .8442 |
| entity marker entity start | 5 |  | .8569 |
| 2sentence-entity-token | 5 |  | .8646 |
| 2-masked-sentence | 5 |  | .8660 |
| Sig. |  | 1.000 | .140 |
| Means for groups in homogeneous subsets are displayed. | | | |
| a. Uses Harmonic Mean Sample Size = 5.000. | | | |

| **precision** | | | |
| --- | --- | --- | --- |
| Tukey HSD^a^ | | | |
| method | N | Subset for alpha = 0.05 | |
|  |  | 1 | 2 |
| default | 5 | .6460 |  |
| masked | 5 |  | .8114 |
| entity marker entity start | 5 |  | .8251 |
| 2-masked-sentence | 5 |  | .8367 |
| 2sentence-entity-token | 5 |  | .8391 |
| Sig. |  | 1.000 | .295 |
| Means for groups in homogeneous subsets are displayed. | | | |
| a. Uses Harmonic Mean Sample Size = 5.000. | | | |

| **recall** | | | |
| --- | --- | --- | --- |
| Tukey HSD^a^ | | | |
| method | N | Subset for alpha = 0.05 | |
|  |  | 1 | 2 |
| default | 5 | .6474 |  |
| masked | 5 |  | .8259 |
| entity marker entity start | 5 |  | .8358 |
| 2sentence-entity-token | 5 |  | .8447 |
| 2-masked-sentence | 5 |  | .8466 |
| Sig. |  | 1.000 | .102 |
| Means for groups in homogeneous subsets are displayed. | | | |
| a. Uses Harmonic Mean Sample Size = 5.000. | | | |

| **fscore** | | | |
| --- | --- | --- | --- |
| Tukey HSD^a^ | | | |
| method | N | Subset for alpha = 0.05 | |
|  |  | 1 | 2 |
| default | 5 | .6419 |  |
| masked | 5 |  | .8165 |
| entity marker entity start | 5 |  | .8281 |
| 2sentence-entity-token | 5 |  | .8396 |
| 2-masked-sentence | 5 |  | .8400 |
| Sig. |  | 1.000 | .101 |
| Means for groups in homogeneous subsets are displayed. | | | |
| a. Uses Harmonic Mean Sample Size = 5.000. | | | |

**D-3. Performance comparison of downstream layers**

| **ANOVA** | | | | | | |
| --- | --- | --- | --- | --- | --- | --- |
|  | | Sum of Squares | df | Mean Square | F | Sig. |
| accuracy | Between Groups | .000 | 2 | .000 | .454 | .646 |
|  | Within Groups | .002 | 12 | .000 |  |  |
|  | Total | .003 | 14 |  |  |  |
| precision | Between Groups | .001 | 2 | .000 | .615 | .557 |
|  | Within Groups | .007 | 12 | .001 |  |  |
|  | Total | .008 | 14 |  |  |  |
| recall | Between Groups | .000 | 2 | .000 | 1.306 | .307 |
|  | Within Groups | .002 | 12 | .000 |  |  |
|  | Total | .002 | 14 |  |  |  |
| fscore | Between Groups | .001 | 2 | .000 | 1.185 | .339 |
|  | Within Groups | .003 | 12 | .000 |  |  |
|  | Total | .003 | 14 |  |  |  |

| **ANOVA Effect Sizes**^a,b^ | | | | |
| --- | --- | --- | --- | --- |
|  | | Point Estimate | 95% Confidence Interval | |
|  |  |  | Lower | Upper |
| accuracy | Eta-squared | .070 | .000 | .311 |
|  | Epsilon-squared | -.085 | -.167 | .196 |
|  | Omega-squared Fixed-effect | -.079 | -.154 | .185 |
|  | Omega-squared Random-effect | -.038 | -.071 | .102 |
| precision | Eta-squared | .093 | .000 | .345 |
|  | Epsilon-squared | -.058 | -.167 | .235 |
|  | Omega-squared Fixed-effect | -.054 | -.154 | .223 |
|  | Omega-squared Random-effect | -.026 | -.071 | .126 |
| recall | Eta-squared | .179 | .000 | .441 |
|  | Epsilon-squared | .042 | -.167 | .348 |
|  | Omega-squared Fixed-effect | .039 | -.154 | .333 |
|  | Omega-squared Random-effect | .020 | -.071 | .199 |
| fscore | Eta-squared | .165 | .000 | .428 |
|  | Epsilon-squared | .026 | -.167 | .332 |
|  | Omega-squared Fixed-effect | .024 | -.154 | .317 |
|  | Omega-squared Random-effect | .012 | -.071 | .188 |
| a. Eta-squared and Epsilon-squared are estimated based on the fixed-effect model. | | | | |
| b. Negative but less biased estimates are retained, not rounded to zero. | | | | |

**Post Hoc Tests**

| **Multiple Comparisons** | | | | | | |
| --- | --- | --- | --- | --- | --- | --- |
| Tukey HSD | | | | | | |
| Dependent Variable | (I) layer | (J) layer | Mean Difference (I-J) | Std. Error | Sig. | 95% Confidence Interval |
|  |  |  |  |  |  | Lower Bound |
| accuracy | CLS-layer | 2-layer | -.00852 | .00904 | .625 | -.0327 |
|  |  | 3-layer | -.00317 | .00904 | .935 | -.0273 |
|  | 2-layer | CLS-layer | .00852 | .00904 | .625 | -.0156 |
|  |  | 3-layer | .00535 | .00904 | .827 | -.0188 |
|  | 3-layer | CLS-layer | .00317 | .00904 | .935 | -.0210 |
|  |  | 2-layer | -.00535 | .00904 | .827 | -.0295 |
| precision | CLS-layer | 2-layer | -.01628 | .01512 | .546 | -.0566 |
|  |  | 3-layer | -.01164 | .01512 | .728 | -.0520 |
|  | 2-layer | CLS-layer | .01628 | .01512 | .546 | -.0241 |
|  |  | 3-layer | .00464 | .01512 | .950 | -.0357 |
|  | 3-layer | CLS-layer | .01164 | .01512 | .728 | -.0287 |
|  |  | 2-layer | -.00464 | .01512 | .950 | -.0450 |
| recall | CLS-layer | 2-layer | -.01216 | .00753 | .277 | -.0322 |
|  |  | 3-layer | -.00640 | .00753 | .680 | -.0265 |
|  | 2-layer | CLS-layer | .01216 | .00753 | .277 | -.0079 |
|  |  | 3-layer | .00576 | .00753 | .731 | -.0143 |
|  | 3-layer | CLS-layer | .00640 | .00753 | .680 | -.0137 |
|  |  | 2-layer | -.00576 | .00753 | .731 | -.0258 |
| fscore | CLS-layer | 2-layer | -.01488 | .00976 | .314 | -.0409 |
|  |  | 3-layer | -.00920 | .00976 | .625 | -.0352 |
|  | 2-layer | CLS-layer | .01488 | .00976 | .314 | -.0111 |
|  |  | 3-layer | .00568 | .00976 | .832 | -.0203 |
|  | 3-layer | CLS-layer | .00920 | .00976 | .625 | -.0168 |
|  |  | 2-layer | -.00568 | .00976 | .832 | -.0317 |

| **Multiple Comparisons** | | | |
| --- | --- | --- | --- |
| Tukey HSD | | | |
| Dependent Variable | (I) layer | (J) layer | 95% Confidence Interval |
|  |  |  | Upper Bound |
| accuracy | CLS-layer | 2-layer | .0156 |
|  |  | 3-layer | .0210 |
|  | 2-layer | CLS-layer | .0327 |
|  |  | 3-layer | .0295 |
|  | 3-layer | CLS-layer | .0273 |
|  |  | 2-layer | .0188 |
| precision | CLS-layer | 2-layer | .0241 |
|  |  | 3-layer | .0287 |
|  | 2-layer | CLS-layer | .0566 |
|  |  | 3-layer | .0450 |
|  | 3-layer | CLS-layer | .0520 |
|  |  | 2-layer | .0357 |
| recall | CLS-layer | 2-layer | .0079 |
|  |  | 3-layer | .0137 |
|  | 2-layer | CLS-layer | .0322 |
|  |  | 3-layer | .0258 |
|  | 3-layer | CLS-layer | .0265 |
|  |  | 2-layer | .0143 |
| fscore | CLS-layer | 2-layer | .0111 |
|  |  | 3-layer | .0168 |
|  | 2-layer | CLS-layer | .0409 |
|  |  | 3-layer | .0317 |
|  | 3-layer | CLS-layer | .0352 |
|  |  | 2-layer | .0203 |

**Homogeneous Subsets**

| **accuracy** | | |
| --- | --- | --- |
| Tukey HSD^a^ | | |
| layer | N | Subset for alpha = 0.05 |
|  |  | 1 |
| CLS-layer | 5 | .8581 |
| 3-layer | 5 | .8612 |
| 2-layer | 5 | .8666 |
| Sig. |  | .625 |
| Means for groups in homogeneous subsets are displayed. | | |
| a. Uses Harmonic Mean Sample Size = 5.000. | | |

| **precision** | | |
| --- | --- | --- |
| Tukey HSD^a^ | | |
| layer | N | Subset for alpha = 0.05 |
|  |  | 1 |
| CLS-layer | 5 | .8232 |
| 3-layer | 5 | .8348 |
| 2-layer | 5 | .8395 |
| Sig. |  | .546 |
| Means for groups in homogeneous subsets are displayed. | | |
| a. Uses Harmonic Mean Sample Size = 5.000. | | |

| **recall** | | |
| --- | --- | --- |
| Tukey HSD^a^ | | |
| layer | N | Subset for alpha = 0.05 |
|  |  | 1 |
| CLS-layer | 5 | .8353 |
| 3-layer | 5 | .8417 |
| 2-layer | 5 | .8474 |
| Sig. |  | .277 |
| Means for groups in homogeneous subsets are displayed. | | |
| a. Uses Harmonic Mean Sample Size = 5.000. | | |

| **fscore** | | |
| --- | --- | --- |
| Tukey HSD^a^ | | |
| layer | N | Subset for alpha = 0.05 |
|  |  | 1 |
| CLS-layer | 5 | .8268 |
| 3-layer | 5 | .8360 |
| 2-layer | 5 | .8416 |
| Sig. |  | .314 |
| Means for groups in homogeneous subsets are displayed. | | |
| a. Uses Harmonic Mean Sample Size = 5.000. | | |
